# An ancient and essential miRNA family controls cellular interaction pathways in *C. elegans*

**DOI:** 10.1126/sciadv.adz1934

**Published:** 2025-09-03

**Authors:** Emilio M. Santillan, Eric D. Cormack, Jingkui Wang, Micaela Rodriguez-Steube, Luisa Cochella

**Affiliations:** ^1^ School of Medicine, Johns Hopkins University, Baltimore, MD, USA.; ^2^ Research Institute of Molecular Pathology (IMP), Vienna BioCenter (VBC), Vienna, Austria.

## Abstract

The transition from unicellular to multicellular life required the acquisition of coordinated and regulated cellular behaviors, including adhesion and migration. In metazoans, this involves adhesion proteins, signaling systems, and an elaborate extracellular matrix (ECM) that contributes to adhesion and signaling interactions. Innovations that enabled complex multicellularity occurred through new genes in these pathways, novel functions for existing genes, and regulatory changes. Gene regulation by microRNAs (miRNAs) expanded with multicellularity. A single miRNA, miR-100, arose in the last common eumetazoan ancestor and is widely conserved across animals. We reveal the molecular function of its *C. elegans* homolog, the miR-51 family. This family acts in a dose-dependent manner to control morphogenesis by regulating several genes involved in cell signaling, adhesion, and migration, including ECM modifiers—specifically heparan sulfate sulfotransferases (HSTs). Some of these targets are also predicted to be conserved targets across vertebrates. Our work suggests that this miRNA provided an innovation in the regulation of cellular interactions early in metazoan evolution.

## Teaser

A microRNA that arose at the origin of eumetazoans regulates cell adhesion and signaling in *C. elegans* through conserved targets.

## INTRODUCTION

**Fig. 1.**
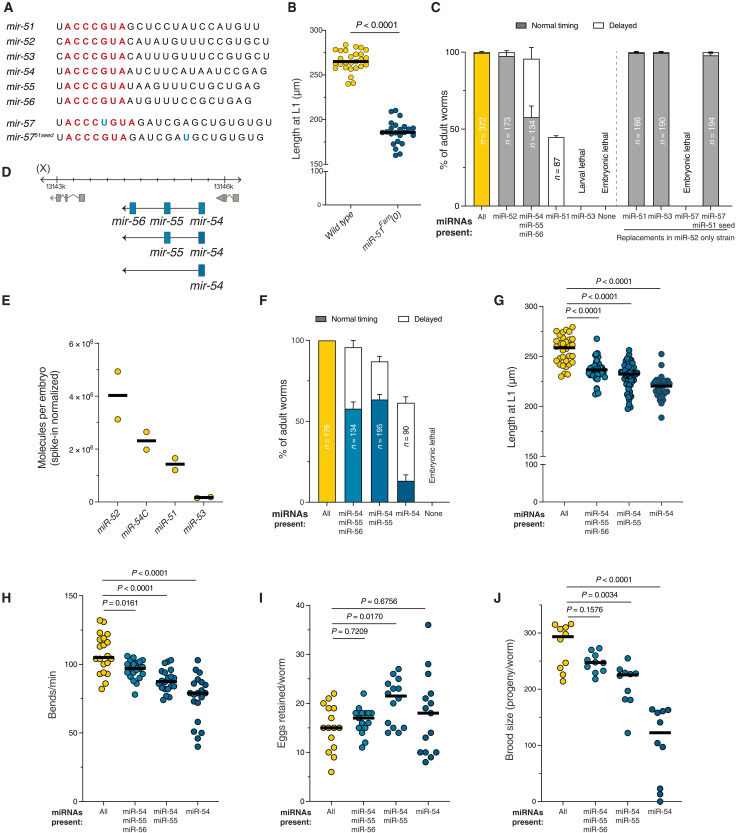
The miR-51 family controls multiple processes in a dose-dependent manner. (**A**) Alignment of the miR-51 family in *C. elegans.* (**B**) Length of L1 larvae at hatching in wild-type (WT) animals or animals with a complete miR-51 family deletion. *P* value from *t* test. Each dot represents one animal. (**C**) Viability measured as the number of animals reaching adulthood for strains in which all but one miR-51 family locus was deleted. Right of dashed line: Viability of animals in which all miR-51 family loci except miR-52 were deleted, but the miR-52 hairpin sequence was replaced by that of the indicated miRNAs. *n*, number of animals scored. Error bars are the SE of the proportion. (**D**) Schematic of the miR-54 cluster and the deletion series generated to isolate the contribution of dose from expression pattern. (**E**) Absolute abundance of each miRNA family member measured by small RNA sequencing using spike-ins. miR-54C denotes the sum of all three cluster members, miR-54, miR-55, and miR-56. (**F**) Viability measured as in (C) for strains with a decreasing dose of miR-51 family. (**G**) Length measured as in (B) for strains with a decreasing dose of miR-51 family. (**H**) Locomotion measured as bends per minute in a liquid drop for strains with a decreasing dose of miR-51 family. (**I**) Egg laying measured as retained eggs per animal for strains with a decreasing dose of miR-51 family. (**J**) Brood size for strains with a decreasing dose of miR-51 family. *P* values in (G) to (J) from analysis of variance (ANOVA) with multiple comparisons correction.

MicroRNAs (miRNAs) provide a layer of posttranscriptional gene repression that is essential for animal development (*1*). The number of miRNAs expanded with various innovations during animal evolution, and extant animals express hundreds of miRNAs (*2*–*6*). About 30 miRNAs are conserved across bilaterians, but a single miRNA, miR-100, has remained conserved across eumetazoans (*6*) (animals with true tissues organized into germ layers, including bilaterians and cnidarians) (fig. S1A). Despite its conservation, the molecular function of miR-100 remains largely unknown. In *Drosophila*, deletion of the single copy of miR-100 does not cause overt defects (*7*). Vertebrates have multiple copies of miR-100 (*5*): There are two in birds, three in mammals, and four in fish (fig. S1B), and no complete deletion of this family in vertebrates has been reported to date. In *Caenorhabditis elegans*, miR-51 is an ortholog of miR-100, and it forms a family with six members (miR-51 through miR-56) that share the seed sequence (nucleotides 2 to 8) and are expressed from four loci (Fig. 1A and fig. S1C). miR-51 is an ortholog of miR-100, while other family members probably arose by convergent evolution (*5*). Deletion of all six family members causes completely penetrant embryonic defects and lethality, which can be rescued by overexpression of any individual member, indicating that they act in a redundant manner (*8*, *9*). We thus used *C. elegans* not only to dissect the molecular function of miR-51/miR-100 but also to understand the need for multiple members of this family.

## RESULTS

Initial characterization of the miR-51 family (miR-51^Fam^) had revealed that complete loss of the family causes embryos to arrest during the elongation stage of morphogenesis, when the body of the worm lengthens to its final shape (*8*, *9*). A failure in attachment of the pharynx to the buccal cavity was also reported (*9*). We confirmed that about half of the animals die as arrested embryos (fig. S1D and movies S1 and S2). We found that the rest die shortly after hatching, and the larvae that do hatch are significantly shorter (Fig. 1B).

The redundancy of the six family members is, however, not complete. Individual loci have a quantitatively different ability to support function as measured by viability and body length [(*8*), Fig. 1C, and fig. S1E]. For example, the miR-52 locus was sufficient to produce fully viable worms of normal length, while animals with all family members deleted except for miR-53 died as larvae that were also short (Fig. 1C and fig. S1E). This differential activity could have three nonexclusive causes: differences in (i) miRNA sequences outside of the seed that may affect target specificity (*10*–*12*), (ii) expression pattern (*13*), and (iii) dose (*14*). To test the contribution of sequences beyond the seed, we took advantage of the strain that lives exclusively on miR-52 and replaced the endogenous miR-52 hairpin by the miR-51 or miR-53 hairpins, using CRISPR-Cas9. These two miRNAs, which are not sufficient to support development from their own loci, provided complete viability when expressed under the miR-52 regulatory sequences (Fig. 1C), and they also fully restored larval length (fig. S1E). When expressed from the miR-52 locus, miR-51 and miR-53 achieved expression levels comparable to miR-52 (fig. S2A). We also replaced miR-52 by miR-57, another ortholog of miR-100 which differs by a single-nucleotide insertion in its seed and is thus not part of the family as it has different targets (Fig. 1A). Introduction of miR-57 did not support viability; however, converting the seed to that of miR-51 was sufficient to fully restore development, including viability and length (Fig. 1, A and C, and fig. S1E). Thus, differences in 3′ sequence alone do not account for functional differences among family members, and the miR-51^Fam^ exerts its function by regulating seed-matched targets.

The severity of the defects observed in the presence of individual miR-51^Fam^ members correlated with the dose of each miRNA, which we measured by small RNA sequencing with spike-ins for absolute quantification (Fig. 1, C and E, and figs. S1E and S2) (*15*, *16*). All family members are broadly expressed [(*9*, *17*) and fig. S1F]. However, because they are expressed from different loci and likely have differences in pattern, we sought to disentangle dosage and pattern directly. To this end, we took advantage of the miR-54 cluster, which produces three miRNAs from a single transcript (−54, −55, and −56), in practically stoichiometric amounts (data S1 and fig. S2B). Starting with a strain that only had the miR-54 cluster (i.e., miR-51, miR-52, and miR-53 deleted), we used CRISPR-Cas9 to generate a deletion series of the cluster members to reduce dose while keeping expression pattern constant (Fig. 1D). We confirmed the progressive decrease in dosage by small RNA sequencing (fig. S2B) and observed that viability and length in these strains follow a dose-dependent relationship with the total amount of miRNA from the miR-51^Fam^ (Fig. 1, F and G). We also tested the effect of increasing the level of the lowly expressed miR-53 in the strain that contains only the miR-53 locus, by injecting extra copies of this locus (including its own promoter) as an extrachromosomal element. Increasing the copy number of miR-53 was sufficient to restore viability and length (fig. S2C). While we cannot exclude that differences in expression pattern of the various members also affect specific functions, we conclude that the miR-51^Fam^ acts in a dose-dependent manner, through seed-matched targets.

While analyzing the deletion series with reduced miRNA dose, we observed that the larvae and adults displayed various pleiotropic phenotypes. Specifically, they had decreased brood sizes, abnormal egg laying causing them to retain eggs in the uterus, and abnormal locomotion, which we quantified in a thrashing assay. All observed phenotypes were also dependent on the miR-51^Fam^ dose (Fig. 1, H to J). These phenotypes do not seem to be caused by loading of nonspecific small RNAs into Argonaute, which could have been expected from an increase of Argonaute:miRNA ratio (*18*, *19*), as the absolute number of non-miRNA mapping small RNAs was not increased in any mutant strain (fig. S2D). The observed defects are also not due to misexpression of other miRNAs as their absolute numbers were unchanged (fig. S2E). Therefore, the dose-dependent function of the miR-51^Fam^ affects diverse organismal processes.

**Fig. 2.**
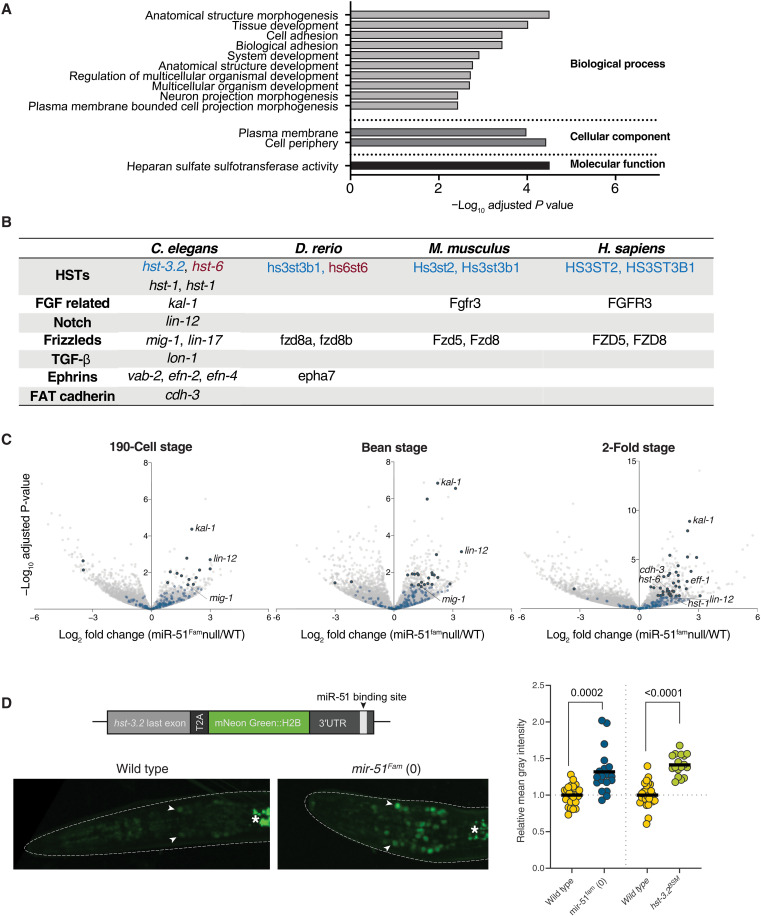
The miR-51 family targets conserved genes involved in signaling and ECM modification. (**A**) GO term enrichment analysis performed on all 293 targets predicted by TargetScanWorm. (**B**) Targets predicted by TargetScan across diverse species. Ortholog pairs are shown in the same color. FGF, fibroblast growth factor; TGF-β, transforming growth factor–β. (**C**) RNA-seq analysis comparison between WT and miR-51 family null embryos at the indicated stages. Targets predicted by TargetScan are in light blue. Predicted targets that are significantly deregulated are in dark blue. (**D**) Target validation strategy: Schematic of the endogenous reporter for *hst-3.2* generated by CRISPR-Cas9, including a nuclear localized fluorophore separated from the protein of interest by a 2A peptide. Targeting was validated by crossing this reporter to the complete miR-51 family deletion and measuring fluorescence. We also introduced mutations in the predicted miR-51 binding sites to test whether they directly control expression of these genes. Representative images and quantification are shown (*P* values from *t* test, each dot is one animal). Arrowheads mark example nuclei with reporter expression; asterisks mark autofluorescence of gut granules.

The functional targets of the miR-51^Fam^ remain unknown. One target has been proposed to be regulated by miR-51^Fam^, the atypical cadherin *cdh-3/FAT4*, but the functional consequence of this regulation is unclear (*9*). To find the targets that are regulated by the miR-51^Fam^ and are responsible for the broad range of observed defects, we first performed a Gene Ontology analysis of the 293 targets predicted by TargetScanWorm 6.2 (*20*), which considers binding site conservation across nematodes. This revealed a strong enrichment for putative targets involved in morphogenesis, acting primarily in the cell membrane or periphery, and with a specific enrichment for heparan sulfate sulfotransferases (HSTs), enzymes involved in modification of heparan sulfate proteoglycans (HSPGs) (Fig. 2A).

Given the deep conservation of miR-51/miR-100 and the fact that other conserved miRNAs have preserved certain targets across long phylogenetic distances [e.g., *let-7* (*21*–*23*) and miR-1 (*24*)], we analyzed conservation of target predictions by TargetScanFish, Mouse and Human (Fig. 2B) (*25*). This revealed that direct orthologs of two types of HSTs are also predicted targets of miR-100 across diverse vertebrates: HS3STs catalyze 3-*O*-sulfation of HSPGs and are predicted targets in worms, fish, and mammals, and HS6STs catalyze 6-*O*-sulfation and are predicted targets in worms and fish. Such conservation is not expected to occur by chance. In addition, we observed that some metazoan-specific signaling pathways that are crucial for development are predicted targets of miR-51/miR-100. For example, miR-51/miR-100 is predicted to target two paralogs of Frizzled receptors, which mediate Wnt signaling, in worms (*mig-1/FZD4* and *lin-17*) and in vertebrates (*FZD5* and *FZD8*) (Fig. 2B).

Three other crucial cell communication pathways for embryonic development are also predicted targets in *C. elegans*. First, three of four ephrin genes in *C. elegans* (*vab-2*/*EFNB1*, *efn-2*/*EFNB3*, and *efn-4*/*EFNB1*) are predicted targets of the miR-51^Fam^ and have been implicated in regulation of morphogenesis (*26*). An ephrin paralog, *epha7*/*EPHA7*/*vab-1*, is also a predicted target in zebrafish (Fig. 2B). Second, *kal-1/ANOS1*, an extracellular protein that has been implicated in cell adhesion and neuronal migration and acts as a coreceptor for fibroblast growth factor (FGF) signaling together with HSPGs (*27*–*29*), is a conserved target of the miR-51^Fam^ in nematodes. KAL-1 also plays roles in cell migration and morphogenesis in parallel to the ephrin/Eph pathway in *C. elegans* (*30*). Third, *lin-12/NOTCH* is one of two Notch receptors in *C. elegans*, a key component of a critical signaling pathway during animal development and a conserved predicted target of the miR-51^Fam^ in nematodes.

To validate these predictions, we performed mRNA sequencing at three defined developmental time points in wild-type and miR-51^Fam^ null embryos. We expected that loss of repression could result in increased mRNA levels of regulated targets (Fig. 2C and fig. S3A). Fifty-one of 293 predicted targets of miR-51 displayed increased mRNA abundance (log fold change > 2, *P* < 0.05), for at least one time point, in embryos lacking the complete miR-51 family (Fig. 2C and data S2). Among these, *hst-1* and *hst-6* were significantly up-regulated. So were *mig-1/FZD4*, *kal-1/ANOS1*, *lin-12/NOTCH*, *cdh-3*/*FAT4* (Fig. 2C, fig. S3B, and data S2). While *hst-3.2* did not reach statistical significance, it also exhibited a trend toward up-regulation in the mutant embryos at all three time points (fig. S3B). This apparent up-regulation was confirmed by introducing a T2A::mNeonGreen::H2B cassette in the endogenous *hst-3.2* locus using CRISPR-Cas9, which allowed us to measure the combined impact of miRNA regulation at the mRNA and protein levels. Expression from this locus was derepressed in miR-51–deficient animals, or upon mutation of the single miR-51 binding site in the *hst-3.2* 3′ untranslated region (3′UTR; Fig. 2D). Therefore, *hst-3.2* is a direct target of the miR-51^Fam^ in *C. elegans.* We also validated that *kal-1/ANOS1* and *lin-12/NOTCH* are direct targets of miR-51 using analogous endogenous reporters generated by CRISPR-Cas9 (fig. S3C). All binding site mutations (BSMs) consisted of precise repair products such that introducing a heterologous miRNA that restores complementarity to the mutant binding site, under the miR-52 promoter, was sufficient to restore repression (fig. S3D). Thus, *kal-1* and *lin-12* are also directly repressed by miR-51^Fam^.

The signaling pathways under miR-51/miR-100 control are metazoan innovations that enable patterning of tissues and organs (*31*). Signaling via these pathways depends on the extracellular matrix (ECM), including HSPGs and their sulfation status (*32*, *33*). It has been proposed that evolution of a metazoan-specific ECM has facilitated the establishment of diffusion gradients that facilitate formation of complex tissues and body plans (*34*). Thus, we hypothesized that regulation of ECM composition and key developmental signaling and adhesion pathways by miR-51/miR-100 could explain the embryonic requirement for this miRNA, as well as the pleiotropic phenotypes in *C. elegans* caused by decreased dose of this miRNA family.

**Fig. 3.**
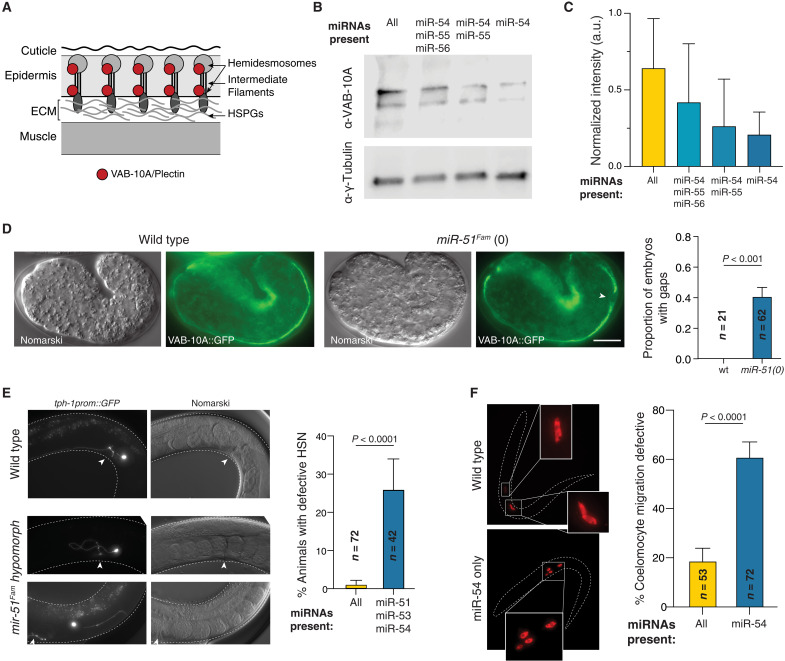
The miR-51 family is necessary for developmental processes that require intercellular communication and adhesion. (**A**) Schematic of the hemidesmosome-like connection between muscle and epidermis that is necessary for successful embryo elongation. (**B**) Western blot using an antibody against VAB-10A on lysates from L4 larvae carrying decreasing dose of miR-51 family. (**C**) Quantification of three independent Western blots. Plotted are mean and SD. a.u., arbitrary units. (**D**) Representative images of VAB-10A::GFP distribution in WT and miR-51^Fam^ null embryos. Arrowhead marks abnormal gap in the otherwise continuous signal. Quantification of such abnormal gaps is shown. Error bars are the SE of the proportion; *P* value from χ^2^ test. (**E**) Representative images of HSN neuron position and projection relative to the vulva (white arrowhead) and quantification of the observed defects in animals with decreased miR-51. The HSN is visualized by expression of the terminal marker *tph-1prom::gfp*. Error bars are the SE of the proportion; *P* value from χ^2^ test. (**F**) Coelomocyte migration and attachment defects visualized by expression of the terminal marker *unc-122prom::mCherry.* Error bars are the SE of the proportion; *P* value from χ^2^ test.

To test this hypothesis, we explored more extensively the role of the miR-51^Fam^ in cell adhesion and signaling. We examined two developmental contexts in which composition of the ECM and activity of key signaling pathways are essential: (i) elongation during morphogenesis (*35*) and (ii) cell migrations during development (*36*, *37*). *C. elegans* elongation during embryogenesis relies on transmission of muscle-generated forces to the epidermis and cuticle via hemidesmosome like structures known as fibrous organelles (FOs), which connect both tissues through the ECM (Fig. 3A) (*38*). We thus examined the integrity of the FO using a well-validated antibody against VAB-10A (*39*), a spectraplakin family protein that is an essential component of this structure and is not a target of miR-51^Fam^. Western blot analysis showed that VAB-10A abundance decreases with the dose of miR-51^Fam^ (Fig. 3, B and C), suggesting a dosage-dependent disruption of connections between muscle and hypodermis. Consistent with this, we observed breaks in the continuity of the connection between these tissues in 25 of 62 (40%) of early embryos both by immunostaining with the same antibody and with a VAB-10::GFP endogenous reporter (Fig. 3D). Weak loss of function alleles of *vab-10* causes elongation defects similar to what we observe (*40*). We found that muscle differentiation and localization in embryos lacking miR-51^Fam^ looked normal, ruling out a failure in force generation (fig. S4). The distribution of the HSPG UNC-52/perlecan in the basal membrane underlying muscle cells also seemed normal (fig. S4), although differences in sulfation status are not expected to affect this. Overall, we conclude that loss of the miR-51^Fam^ most likely causes a defect in muscle and epidermis attachment that could explain, at least in part, the observed elongation failure during embryogenesis. This defect is reminiscent of that observed in *C. elegans* embryos lacking the two miRNA-loading Argonaute proteins (*41*), consistent with our previous observation that the miR-51^Fam^ explains a large part of the requirement for miRNAs in the *C. elegans* embryo (*16*).

The second developmental process in which the importance of signaling gradients and ECM composition have been well documented is cell migration (*36*, *37*). We first examined the migration and axonal morphology of the neurons that innervate the vulva muscles, the hermaphrodite-specific neurons (HSNs). These neurons are born in the tail of the animal and migrate during embryogenesis to the mid-body (*42*). They later innervate the vulva and send a projection to the head where they connect with the nerve ring, ultimately controlling egg laying (*42*). HSN development is influenced by a variety of genetic pathways including cell signaling proteins and axon guidance mechanisms (e.g., MIG-1/Frizzled, UNC-6/Netrin, UNC-40/DCC, and others) (*42*) and the modification of HSPGs (*43*–*46*). We observed that even partial loss of the miR-51^Fam^ caused severe migration and axon pathfinding defects in the HSNs (Fig. 3E). We also examined the migration of another cell type that is unrelated to the HSNs both in terms of function and lineage origin: two coelomocyte mothers migrate during embryogenesis to occupy stereotypic positions and divide symmetrically to give rise to two coelomocyte pairs that remain attached or very close to one another. Coelomocyte migration is also dependent on HSPGs (*43*). We observed that with a decreased dose of the miR-51^Fam^, the coelomocytes fail to reach their stereotypic positions, and the members of each pair detach from one another (Fig. 3F). In conclusion, the miR-51^Fam^ is necessary for various developmental processes that depend on regulated cell adhesion and signaling.

To directly link the deregulation of specific targets to the observed phenotypes, we introduced binding site mutations (BSMs) in 12 targets involved in signaling and ECM modification introduced above (Fig. 2B and fig. S5A). We also included *eff-1* as a high priority candidate, as it is significantly derepressed upon loss of miR-51^Fam^ (fig. S3B), it mediates various membrane remodeling events (*47*), and it physically interacts with VAB-10A/spectraplakin (*48*). The expectation is that if deregulation of these targets is responsible for the observed defects upon loss of miR-51^Fam^, removing the miR-51 binding sites in their 3′UTRs (in an otherwise miR-51^Fam^ wild-type background) should result in the same defects. We generated all BSMs using CRISPR-Cas9 in a wild-type background, and in each case selected for precise repair products that changed the miR-51 binding sites to generate a binding site for *lsy*-6, a miRNA that is expressed in a single *C. elegans* neuron and whose biology is very well understood (fig. S5B) (*49*). This would enable a later rescue experiment of any potential defect by expressing *lsy-6* under the miR-52 promoter (fig. S5B). We first generated two strains with three BSMs each (3xBSM), one with all the ephrins mutated (*vab-2*, *efn-2*, and *efn-4*) and another containing BSMs in *hst-3.2*, *hst-6*, and *kal-1*. We crossed these two to generate a strain with BSMs in six target genes (6x). Because of constrains imposed by linkage of the various targets, we then performed sequential CRISPR-Cas9 edits to introduce BSMs in *eff-1* (7x), *hst-1*/*NDST* (8x), *hst-2*/*HS2ST1* (9x), *lon-1* (10x), *lin-12/NOTCH* (11x), *mig-1*/*FZD4* (12x), and, lastly, *cdh-3*/*FAT4* (13x).

**Fig. 4.**
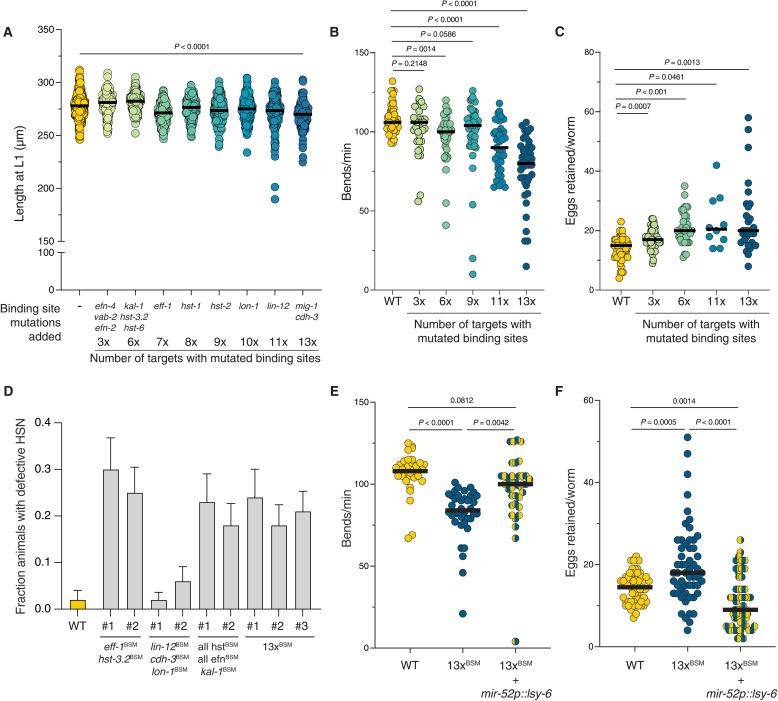
Multiple targets cumulatively explain miR-51 family function. (**A**) Length at L1 measured in the series of strains with progressive addition of BSMs. The names of the genes below each dataset indicate those in which BSMs were added relative to the previous dataset, e.g., the 7x strain has BSMs in *efn-4*, *vab-2*, *efn-2*, *kal-1*, *hst-3.2*, *hst-6*, and *eff-1. P* values in all panels from ANOVA with multiple comparisons correction. In all plots, each dot represents one animal. (**B**) Locomotion measured in the series of strains with progressive addition of BSMs. The numbers correspond to the same genotypes of the strains shown in (A). (**C**) Egg laying measured in the series of strains with progressive addition of BSMs. The numbers correspond to the same strains shown in (A). (**D**) HSN migration and axon pathfinding defects in strains with diverse combinations of BSMs obtained by outcrossing the 13x strain with a strain containing the *tph-1prom::gfp* reporter used to visualize the HSN. We recovered strains with the indicated genes containing BSMs and all others WT. Error bars represent the SE of the proportion. *n* > 45 animals per genotype. (**E**) Rescue of the locomotion defect observed in the 13xBSM strain by transgenic expression of a miRNA that binds the mutant binding sites, under the miR-52 promoter. (**F**) Rescue of the egg laying defect observed in the 13xBSM strain by transgenic expression of a miRNA that binds the mutant binding sites, under the miR-52 promoter.

We found that whereas these targets explain only a fraction of the short length of the animals at hatching (Fig. 4A, compare to Fig. 1), the derepression of multiple targets cumulatively phenocopied the defects in locomotion and egg laying that we saw upon loss of miR-51^Fam^ [Fig. 4 (B and C), compare to Fig. 1 (H and I)]. Because of the way these strains were constructed, we could not assess the contribution of each individual target or the large number of possible target combinations. However, we attempted to further dissect the contributions of subsets of targets by outcrossing the 13xBSM strain and recovering different mutant combinations (which was somewhat limited by linkage; fig. S5A). We separated a few different mutant combinations and measured egg retention (fig. S5C) and locomotion (fig. S5D), but we also crossed in an HSN marker to be able to score HSN migration. We observed that derepression of selected targets also phenocopied the HSN migration defects (Fig. 4D). This analysis also revealed that although addition of BSMs in *lin-12/NOTCH* and *cdh-3/FAT4*, to generate the 11x and 13xBSM strains, contributed to the different measured phenotypes, they are not sufficient to cause the observed defects when outcrossed from the 13xBSM strain (Fig. 4D and fig. S5, C and D). This means that it is the combined derepression of multiple targets, including but not limited to these 13 genes, that explains the defects observed upon loss of the miR-51^Fam^. We thus established a causal relationship between small effect increases in gene expression and large effect organismal defects.

We could confirm that the observed phenotypes are specifically caused by the introduced BSMs and not by other mutations that may have occurred during the various gene editing rounds. Expression of *lsy-6*, the miRNA that binds to the mutated binding sites in all 13 targets (fig. S5B), under the miR-52 promoter, was sufficient to rescue the locomotion and egg laying defects (Fig. 4, E and F).

## DISCUSSION

In sum, our work shows that the miR-51/miR-100 miRNA family is required for regulation of multiple targets involved in cell-cell interactions. miRNA-mediated regulation of ECM components or their modifiers appears to be an advantageous evolutionary strategy. Multiple enzymes involved in glycosylation are predicted targets of miRNAs (*50*). The vertebrate-specific miR-223 controls N-glycan biosynthesis in endothelial cells in zebrafish, where it is necessary for differentiation of hematopoietic stem cells, likely through the regulation of signaling pathways such as Notch (*51*). Moreover, loss of miR-79 in *C. elegans* results in a strong reduction in HSPG biosynthesis, and thus neuronal migration defects, by regulating enzymes involved in early proteoglycan biogenesis (*44*).

The dose-dependent function of the miR-51^Fam^ argues that one or more targets are exquisitely dosage sensitive. Several signaling pathways display Goldilocks effects with too much or too little signaling being detrimental. Adhesion forces during development also need to be finely tuned, for example, during worm elongation (*38*). The Kallmann syndrome protein ANOS1/KAL-1, one of the targets we validated in this study, seems to be dosage sensitive in worms and humans. In *C. elegans*, both loss of function of *kal-1* and its transgenic overexpression (although at levels higher than those induced by loss of miR-51) cause neuronal wiring defects that are dependent on HSPG sulfation (*27*–*29*). In humans, loss of function of ANOS1 causes neuronal migration defects that result in Kallmann syndrome, but a microduplication that causes overexpression of ANOS1 also seems to cause disease (*52*). Our work suggests that targets of the miR-100/miR-51 family are dosage-sensitive genes.

The dosage sensitivity of the miR-51^Fam^ also provides an opportunity for evolutionary diversification of gene regulation. Given the expression of family members from different loci, it is possible that tissue- or stage-specific modulation of total miR-51^Fam^ miRNA levels contributes to variation in target repression in a context-specific manner. Moreover, variation of number and strength of miRNA binding sites could result in increasingly complex patterns of regulation. Future studies focusing on the spatial, temporal, and interspecies variation in target sites could further illuminate the developmental function and evolutionary impact of this deeply conserved miRNA family.

The targets we have identified explain a substantial fraction but not the complete set of phenotypes caused by loss of the miR-51 family. Specifically, whereas we see a trend for impaired elongation in the 13xBSM strain (Fig. 4A), we do not observe the severe embryonic morphogenesis defect present upon loss of the complete family (Fig. 1B, fig. S1D, and movie S2). We considered the possibility that more severe defects could be indirect. Specifically, due to gain of function of other miRNAs or other small RNAs as a consequence of the loss of an abundant miRNA family, given that an excess of empty Argonaute could result in loading of aberrant small RNAs (*19*). However, this is unlikely to explain the miR-51 mutant defects as absolute quantification of small RNAs did not reveal changes in the abundance of other miRNAs or of non-miRNA–derived small RNAs (fig. S2, D and E). We thus conclude that other functionally relevant, direct targets of the miR-51 family remain to be identified.

Defects caused by overexpression of a gene are generally interpreted as gain of function due to excess of the gene’s wild-type activity, either in their endogenous context or due to gain of ectopic expression. However, increased protein expression can also cause loss-of-function phenotypes. For example, derepression of V–adenosine triphosphatase (ATPase) subunits upon loss of miR-1 causes loss of function of the V-ATPase complex due to assembly defects (*24*). Our findings provide a paradigm to further study how seemingly modest changes in gene expression caused by miRNA deletions (as measured by mRNA sequencing and endogenous reporters) can result in substantial cellular and organismal defects.

Known essential miRNAs for animal embryogenesis—miR-430 in zebrafish (*53*), miR-35 and miR-51 in *C. elegans* (*8*, *9*) and miR-305 and miR-290 in mice (*54*)—occur in multimembered families, many of them reaching very high levels in the embryo. In contrast to miRNA families like *let-7*, which are composed of members that have acquired different targeting specificities and thus distinct functions (*55*), our data suggest that achieving high dose explains a large part of the need for miR-51 family expansion. A high expression level may be required to achieve sufficient concentration in the rather large early embryonic cells in some animals (*56*). The need for high dose may also be driven by the existence of multiple targets or few targets present at high concentration.

The predicted conservation of targets in other organisms suggests that the function of miR-51^Fam^ has been conserved across distantly related metazoans. Consistent with this model, expression data in human cell lines indicate that the miR-100 family is predominantly expressed in cell types associated with connective tissue and of mesenchymal origin (fig. S6A) (*57*). We propose that miR-51/miR-100 acquired its function early during the evolution of complex multicellular organisms. Our findings provide insight into critical cellular processes whose quantitative regulation has an impact on tissue and organismal morphogenesis and will guide the exploration of the function of this ancient animal miRNA in other animals.

## MATERIALS AND METHODS

### *C. elegans* strain maintenance

All *C. elegans* strains were maintained at 20°C on nematode growth media (NGM) plates seeded with *Escherichia coli* OP50 bacteria as a food source, unless indicated otherwise (*58*).

### Strain generation

For genome engineering, worms were subjected to Cas9-mediated genome engineering via ribonucleoprotein microinjection as described in (*59*). Briefly, purified recombinant Cas9 (4 μg/μl) was coinjected in the gonads of 1-day-old adult worms along with a mixture containing 300 mM KCl, 20 mM Hepes, tracerRNA (500 ng/μl), and coinjection marker *myo-2p::mCherry* (100 ng/μl). In addition, the mix contained CRISPR RNA (100 ng/μl; Integrated DNA Technologies) targeting the locus of interest, as well as double-stranded DNA or single-stranded DNA repair template (50 to 200 ng/μl), harboring the desired modifications and >35-bp flanking homology arms. Injected worms were transferred to bacteria-seeded NGM plates, and 2 days postinjection, *myo-2p::mCherry*-positive progeny were singled onto fresh plates. These were genotyped for the desired modifications after laying progeny.

### Microscopy

For phenotypic analysis and/or quantification of fluorescent reporters, animals were mounted on thin agarose pads on a microscope slide and sealed with a coverslip and vaseline/transparent nail polish. Depending on the magnification and resolution required, two microscopes were mainly used in this work: a Zeiss Axio Imager Z2 microscope equipped with differential interference contrast microscopy (Nomarski) and epifluorescence optics, for lower magnification/resolution or a Zeiss Axio Observer microscope equipped with a CSU-W1 SoRA spinning disk scan head (Yokogawa) and an iXon Life 888 EMCCD camera, when higher resolution was required.

### Fluorescence quantification

Image analysis for fluorescence quantification was performed using Fiji/ImageJ software. A region of interest (ROI) was defined in each case, and the total fluorescence was quantified across the *Z* axis via a sum of slices Z project stack. The background fluorescence was subtracted for each image either by direct subtraction of an “only-background” ROI in the same picture or via the rolling ball method (*60*) when background fluorescence was not uniform. Background uniformity was assessed by plotting gray value intensity profiles along linear ROIs in both the *X* and *Y* directions. To avoid affecting signal, the radius of the rolling ball was set to approximately twice the size of the objects of interest (e.g., twice the diameter of embryonic nuclei). In all cases, fluorescence was normalized to the mean fluorescence of the control condition.

### Phenotypic measurements

#### Length

One-day-old adult worms were dissected using a razor blade to extract embryos, which were then washed twice with M9 buffer. The embryos were incubated for 24 hours at 20°C in M9 buffer to allow hatching and synchronization at the L1 stage. Following incubation, the L1 larvae were immobilized by transferring them to a 20-μl drop of 200 mM sodium azide on a glass slide. Immobilized larvae were then mounted onto agarose pads (2%) for imaging. All images were captured using a Zeiss Axio Imager Z2 microscope with a 20× objective. Larval length was measured using Fiji software (ImageJ) for image analysis.

#### Viability

One-day-old adult worms were dissected with a razor blade to extract embryos. Approximately 30 embryos were transferred in small groups to fresh NGM plates seeded with *E. coli* OP50 as a food source. After 24 hours, the number of unhatched embryos (nonviable progeny) was recorded. After 48 hours, L4 larvae were counted to determine the number of viable progenies. Plates were also screened for unviable larvae at this time. Any L4 larvae observed after 48 hours were classified as viable delayed progeny.

#### Thrashing

One-day-old adult worms were transferred into a glass slide with a depression well containing M9 buffer. Locomotion was assessed by counting the number of body bends made in a 1-min period. A body bend was defined as a complete crossing of the worm’s body midline, followed by a return to the original position.

#### Egg retention

One-day-old adult worms were transferred into 10-μl drops of 4% sodium hypochlorite solution on a glass slide. Once the worms fully dissolved, the number of retained embryos was counted.

#### Brood size

Approximately 10 L4-stage worms were individually transferred to NGM plates seeded with *E. coli* OP50 as a food source. Worms were transferred to fresh plates daily for a total of 4 days. The number of viable progenies on each plate was counted 2 days after the removal of the adult worm, and the total number of viable progenies per worm was summed across all plates.

#### HSN migration and axon routing

One-day-old adults expressing a *tph-1::gfp* reporter, either from an integrated strain (miR-51 mutant strain) or from an extrachromosomal array (BSM strains), were immobilized in a 20-μl drop of 200 mM sodium azide on a glass slide and then mounted onto 2% agarose pads for imaging. HSN migration and axon development were assessed via epifluorescence using a Zeiss Axio Imager Z2 microscope with a 20× or 63× objective.

#### Coelomocyte migration

One-day-old adults expressing an *unc-122prom::mCherry* reporter from an extrachromosomal array were dissected with a razor blade to extract embryos. The embryos were incubated overnight at 20°C in M9 buffer to allow hatching and synchronization at the L1 stage. L1 larvae were immobilized in a 20-μl drop of 200 mM sodium azide on a glass slide and mounted onto 2% agarose pads for imaging. Coelomocytes migration was assessed via epifluorescence using a Zeiss Axio Imager Z2 microscope with a 20× objective. Migration was categorized as normal (wild type) or defective, based on the position of coelomocytes within the larva’s body. Representative images were captured using a Hamamatsu ORCA-Flash4.0 LT3 complementary metal-oxide semiconductor camera.

### Small RNA sequencing and data analysis

Small RNA library preparation and analysis were done as in (*16*). Briefly, samples containing 50 embryos (derived from 1-day-old adults) were collected at the comma stage and immediately snap-frozen in liquid nitrogen. For normalization, a series of eight RNA spike-in oligos (*15*) spanning a 500-fold concentration range was added on a per embryo basis, and total RNA was extracted using TRIzol reagent (Invitrogen) according to the manufacturer’s recommendations for cell samples. Small RNAs (<200 nt) were enriched using the Zymo Research RNA Clean and Concentrator-5 kit (catalog no. R1016). Barcoded 3′ adapters (srBC) were ligated to small RNAs in an overnight reaction at 16°C. On the following day, the ligated products were polyacrylamide gel electrophoresis (PAGE)–purified and eluted using the Zymo Research PAGE elution kit. A 5′ adapter was then ligated overnight at 16°C. Reactions were purified using magnetic beads and reverse-transcribed with SuperScript II. The resulting cDNA libraries were amplified with PCR performed using the TruSeq Universal Adapter and barcoded TruSeq Index reverse primers (KAPA Real-Time Library Amplification kit). Amplification was monitored using fluorescent standards. The final libraries were size-selected on a 3% agarose gel and extracted using the Zymoclean Gel DNA recovery kit. Libraries were multiplexed, and sequencing was performed on an Illumina HiSeqV4 machine.

Small RNA reads were mapped and processed as in (*16*). Briefly, both mapped sequences and associated random nucleotides [functioning as unique molecular identifiers (UMIs)] from the raw reads were quantified for miRNAs and Piwi-interacting RNAs (piRNAs) as well as spike-ins with the Nextflow pipeline in Zenodo (10.5281/zenodo.15883590) and GitHub (https://github.com/lengfei5/smallRNA_nf/tree/master/dev_sRBC). To accurately quantify small RNAs, to alleviate PCR amplification bias, UMI counts were used and normalized with spike-in (attomoles per μg of total RNA) as described in (*15*). Alternatively, as piRNAs are not affected across conditions and can thus be considered an internal control, and data were normalized in parallel using the total number of piRNA reads, yielding results highly similar to the ones by spike-in normalization.

### mRNA sequencing and data analysis

Samples containing 50 embryos (derived from 1-day-old adults) were collected at the two-cell stage and let to develop in M9 buffer until the desired developmental time point. Samples were then snap-frozen in liquid nitrogen and total RNA was extracted using TRIzol reagent (Invitrogen) according to the manufacturer’s recommendations for cell samples. For mRNA library preparation, we used the Lexogen QuantSeq 3′ mRNA-Seq Library Prep Kit, following manufacturer’s recommendations for low-input conditions. Briefly, total RNA was reverse-transcribed using oligo dT priming to capture polyadenylated mRNA, followed by second-strand synthesis. UMIs were incorporated to track individual transcripts and minimize amplification bias. Libraries were amplified with barcodes incorporated in both i5 and i7 primers, enabling multiplexing. Amplification of libraries was monitored using fluorescent standards. Final libraries were purified and sequenced on an Illumina platform

The gene expression profiles by QuantSeq were processed and analyzed in the same way as in (*24*). Briefly, UMIs were first extracted with UMI tools (*61*). Adaptors and first 4 bp of low quality of raw reads were trimmed, and reads were then mapped to the *C. elegans* genome (WBcel235) using a STAR aligner (*62*) with the parameter max_IntronL = 50000. Duplicated mapped reads were discarded with UMI tools, and UMI counts were quantified using HTSeq count (*63*). Differential expression between conditions was analyzed using the DESeq2 package (*64*).

### Western blotting

To assess the levels of FO component VAB-10A, 100 L4s of the desired genotypes were washed three times in ice-cold M9 buffer and then transferred into 35 μl of radioimmunoprecipitation assay buffer. The samples were homogenized through 5 cycles of snap freezing in liquid nitrogen, 5-min incubation at 65°C, and 10-s vortexing. Laemmli solution was added after homogenization, and the samples were then denatured at 95°C and loaded in a q 10-well precast SDS-PAGE gel. Total Proteins were separated via SDS-PAGE and transferred onto a nitrocellulose membrane with a Bio-Rad Trans-Blot SD. The membrane was blocked in a 5% milk/1% Tween solution in tris-buffered saline buffer (TBST) for 1 hour at room temperature. Primary antibody incubation was performed overnight at 4°C. The antibody used was anti–vab-10 [MH5 monoclonal, obtained from Developmental Studies Hybridoma Bank (DSHB), 0.2 μg/ml in 5% milk in TBST]. Following three 10-min washes in TBST, the membrane was incubated with secondary antibody [anti-mouse–horseradish peroxidase (HRP), Cell Signaling 7076, 1:2000 in 5% milk/TBST]. For detection, the membrane was treated with Thermo Fisher Scientific SuperSignal West Femto Maximum Sensitivity Substrate, which is an ultrasensitive enhanced chemiluminescent substrate for low femtogram protein level detection with an HRP enzyme. Imaging of the membrane was done in a Bio-Rad ChemiDoc machine.

### Statistical analysis

Unless otherwise specified, statistical analysis was performed using an unpaired two-tailed *t* test for direct comparisons between two conditions or using a one-way analysis of variance (ANOVA) followed by multiple comparisons by Tukey for when more than two conditions were compared. In all these cases, a normal distribution and homoscedasticity was tested, and residuals plots were analyzed.

## Supplementary Materials

### The PDF file includes:

Figs. S1 to S6 Table S1 Legends for movies S1 and S2 Legends for data S1 and S2 References

## References

[1] C. Alberti, L. Cochella, A framework for understanding the roles of miRNAs in animal development. Development 144, 2548–2559 (2017). 28720652 10.1242/dev.146613

[2] L. F. Sempere, C. N. Cole, M. A. McPeek, K. J. Peterson, The phylogenetic distribution of metazoan microRNAs: Insights into evolutionary complexity and constraint. J. Exp. Zool. B Mol. Dev. Evol. 306, 575–588 (2006). 16838302 10.1002/jez.b.21118

[3] A. M. Heimberg, A. M. Heimberg, L. F. Sempere, L. F. Sempere, V. N. Moy, V. N. Moy, P. C. J. Donoghue, P. C. J. Donoghue, K. J. Peterson, K. J. Peterson, MicroRNAs and the advent of vertebrate morphological complexity. Proc. Natl. Acad. Sci. U.S.A. 105, 2946–2950 (2008). 18287013 10.1073/pnas.0712259105 PMC2268565

[4] A. Grimson, M. Srivastava, B. Fahey, B. J. Woodcroft, H. R. Chiang, N. King, B. M. Degnan, D. S. Rokhsar, D. P. Bartel, Early origins and evolution of microRNAs and Piwi-interacting RNAs in animals. Nature 455, 1193–1197 (2008). 18830242 10.1038/nature07415 PMC3837422

[5] B. Fromm, D. Domanska, E. Høye, V. Ovchinnikov, W. Kang, E. Aparicio-Puerta, M. Johansen, K. Flatmark, A. Mathelier, E. Hovig, M. Hackenberg, M. R. Friedländer, K. J. Peterson, MirGeneDB 2.0: The metazoan microRNA complement. Nucleic Acids Res. 48, D132–D141 (2020). 31598695 10.1093/nar/gkz885 PMC6943042

[6] A. W. Clarke, E. Høye, A. A. Hembrom, V. M. Paynter, J. Vinther, Ł. Wyrożemski, I. Biryukova, A. Formaggioni, V. Ovchinnikov, H. Herlyn, A. Pierce, C. Wu, M. Aslanzadeh, J. Cheneby, P. Martinez, M. R. Friedländer, E. Hovig, M. Hackenberg, S. U. Umu, M. Johansen, K. J. Peterson, B. Fromm, MirGeneDB 3.0: Improved taxonomic sampling, uniform nomenclature of novel conserved microRNA families and updated covariance models. Nucleic Acids Res. 53, D116–D128 (2025). 39673268 10.1093/nar/gkae1094 PMC11701709

[7] N. S. Sokol, P. Xu, Y. N. Jan, V. Ambros, Drosophila let-7 microRNA is required for remodeling of the neuromusculature during metamorphosis. Genes Dev. 22, 1591–1596 (2008). 18559475 10.1101/gad.1671708 PMC2428057

[8] E. Alvarez-Saavedra, H. R. Horvitz, Many families of *C. elegans* microRNAs are not essential for development or viability. Curr. Biol. 20, 367–373 (2010). 20096582 10.1016/j.cub.2009.12.051 PMC2844791

[9] W. R. Shaw, J. Armisen, N. J. Lehrbach, E. A. Miska, The conserved miR-51 microRNA family is redundantly required for embryonic development and pharynx attachment in *Caenorhabditis elegans*. Genetics 185, 897–905 (2010). 20421599 10.1534/genetics.110.117515 PMC2900971

[10] J. Brennecke, A. Stark, R. B. Russell, S. M. Cohen, Principles of microRNA–target recognition. PLoS Biol. 3, e85 (2005). 15723116 10.1371/journal.pbio.0030085 PMC1043860

[11] A. Grimson, K. K.-H. Farh, W. K. Johnston, P. Garrett-Engele, L. P. Lim, D. P. Bartel, MicroRNA targeting specificity in mammals: Determinants beyond seed pairing. Mol. Cell 27, 91–105 (2007). 17612493 10.1016/j.molcel.2007.06.017 PMC3800283

[12] J. P. Broughton, M. T. Lovci, J. L. Huang, G. W. Yeo, A. E. Pasquinelli, Pairing beyond the seed supports microRNA targeting specificity. Mol. Cell 64, 320–333 (2016). 27720646 10.1016/j.molcel.2016.09.004 PMC5074850

[13] R. J. Parchem, J. Ye, R. L. Judson, M. F. LaRussa, R. Krishnakumar, A. Blelloch, M. C. Oldham, R. Blelloch, Two miRNA clusters reveal alternative paths in late-stage reprogramming. Cell Stem Cell 14, 617–631 (2014). 24630794 10.1016/j.stem.2014.01.021 PMC4305531

[14] K. Chen, N. Rajewsky, The evolution of gene regulation by transcription factors and microRNAs. Nat. Rev. Genet. 8, 93–103 (2007). 17230196 10.1038/nrg1990

[15] S. Lutzmayer, B. Enugutti, M. D. Nodine, Novel small RNA spike-in oligonucleotides enable absolute normalization of small RNA-Seq data. Sci. Rep. 7, 5913 (2017). 28724941 10.1038/s41598-017-06174-3 PMC5517642

[16] P. J. Dexheimer, J. Wang, L. Cochella, Two microRNAs are sufficient for embryonic patterning in *C. elegans*. Curr. Biol. 30, 5058–5065.e5 (2020). 33125867 10.1016/j.cub.2020.09.066 PMC7758728

[17] N. J. Martinez, M. C. Ow, J. S. Reece-Hoyes, M. I. Barrasa, V. R. Ambros, A. J. M. Walhout, Genome-scale spatiotemporal analysis of *Caenorhabditis elegans* microRNA promoter activity. Genome Res. 18, 2005–2015 (2008). 18981266 10.1101/gr.083055.108 PMC2593583

[18] N. J. Martinez, R. I. Gregory, Argonaute2 expression is post-transcriptionally coupled to microRNA abundance. RNA 19, 605–612 (2013). 23485552 10.1261/rna.036434.112 PMC3677276

[19] B. Reichholf, V. A. Herzog, N. Fasching, R. A. Manzenreither, I. Sowemimo, S. L. Ameres, Time-resolved small RNA sequencing unravels the molecular principles of microRNA homeostasis. Mol. Cell 75, 756–768.e7 (2019). 31350118 10.1016/j.molcel.2019.06.018 PMC6713562

[20] B. P. Lewis, C. B. Burge, D. P. Bartel, Conserved seed pairing, often flanked by adenosines, indicates that thousands of human genes are microRNA targets. Cell 120, 15–20 (2005). 15652477 10.1016/j.cell.2004.12.035

[21] B. J. Reinhart, F. J. Slack, M. Basson, A. E. Pasquinelli, J. C. Bettinger, A. E. Rougvie, H. R. Horvitz, G. Ruvkun, The 21-nucleotide let-7 RNA regulates developmental timing in *Caenorhabditis elegans*. Nature 403, 901–906 (2000). 10706289 10.1038/35002607

[22] B. R. M. Schulman, A. Esquela-Kerscher, F. J. Slack, Reciprocal expression of lin-41 and the microRNAs let-7 and mir-125 during mouse embryogenesis. Dev. Dyn. 234, 1046–1054 (2005). 16247770 10.1002/dvdy.20599 PMC2596717

[23] Y.-C. Lin, L.-C. Hsieh, M.-W. Kuo, J. Yu, H.-H. Kuo, W.-L. Lo, R.-J. Lin, A. L. Yu, W.-H. Li, Human TRIM71 and its nematode homologue are targets of let-7 microRNA and its zebrafish orthologue is essential for development. Mol. Biol. Evol. 24, 2525–2534 (2007). 17890240 10.1093/molbev/msm195

[24] P. Gutiérrez-Pérez, E. M. Santillán, T. Lendl, J. Wang, A. Schrempf, T. L. Steinacker, M. Asparuhova, M. Brandstetter, D. Haselbach, L., Cochella, miR-1 sustains muscle physiology by controlling V-ATPase complex assembly. Sci. Adv. 7, eabh1434 (2021). 34652942 10.1126/sciadv.abh1434 PMC8519577

[25] V. Agarwal, G. W. Bell, J.-W. Nam, D. P. Bartel, Predicting effective microRNA target sites in mammalian mRNAs. eLife 4, 101 (2015). 10.7554/eLife.05005 PMC4532895 26267216

[26] J. S. Simske, J. Hardin, Getting into shape: Epidermal morphogenesis in *Caenorhabditis elegans* embryos. Bioessays 23, 12–23 (2001). 11135305 10.1002/1521-1878(200101)23:1<12::AID-BIES1003>3.0.CO;2-R

[27] E. I. Rugarli, E. Di Schiavi, M. A. Hilliard, S. Arbucci, C. Ghezzi, A. Facciolli, G. Coppola, A. Ballabio, P. Bazzicalupo, The Kallmann syndrome gene homolog in *C. elegans* is involved in epidermal morphogenesis and neurite branching. Development 129, 1283–1294 (2002). 11874923 10.1242/dev.129.5.1283

[28] H. E. Bülow, K. L. Berry, L. H. Topper, E. Peles, O. Hobert, Heparan sulfate proteoglycan-dependent induction of axon branching and axon misrouting by the Kallmann syndrome gene kal-1. Proc. Natl. Acad. Sci. U.S.A. 99, 6346–6351 (2002). 11983919 10.1073/pnas.092128099 PMC122951

[29] C. A. Díaz-Balzac, M. I. Lázaro-Peña, G. A. Ramos-Ortiz, H. E. Bülow, The adhesion molecule KAL-1/anosmin-1 regulates neurite branching through a SAX-7/L1CAM-EGL-15/FGFR receptor complex. Cell Rep. 11, 1377–1384 (2015). 26004184 10.1016/j.celrep.2015.04.057 PMC4464948

[30] M. L. Hudson, T. Kinnunen, H. N. Cinar, A. D. Chisholm, *C. elegans* Kallmann syndrome protein KAL-1 interacts with syndecan and glypican to regulate neuronal cell migrations. Dev. Biol. 294, 352–365 (2006). 16677626 10.1016/j.ydbio.2006.02.036

[31] A. Sebé-Pedrós, B. M. Degnan, I. Ruiz-Trillo, The origin of Metazoa: A unicellular perspective. Nat. Rev. Genet. 18, 498–512 (2017). 28479598 10.1038/nrg.2017.21

[32] X. Lin, Functions of heparan sulfate proteoglycans in cell signaling during development. Development 131, 6009–6021 (2004). 15563523 10.1242/dev.01522

[33] E. Tecle, C. A. Diaz-Balzac, H. E. Bülow, Distinct 3-O-sulfated heparan sulfate modification patterns are required for kal-1-dependent neurite branching in a context-dependent manner in *Caenorhabditis elegans*. G3 3, 541–552 (2013). 23451335 10.1534/g3.112.005199 PMC3583460

[34] L. S. Babonis, M. Q. Martindale, Phylogenetic evidence for the modular evolution of metazoan signalling pathways. Philos. Trans. R. Soc. B Biol. Sci. 372, 20150477 (2017). 10.1098/rstb.2015.0477 PMC5182411 27994120

[35] M. Labouesse, Role of the extracellular matrix in epithelial morphogenesis: A view from *C. elegans*. Organogenesis 8, 65–70 (2012). 22692230 10.4161/org.20261 PMC3429514

[36] D. C. Merz, G. Alves, T. Kawano, H. Zheng, J. G. Culotti, UNC-52/perlecan affects gonadal leader cell migrations in *C. elegans* hermaphrodites through alterations in growth factor signaling. Dev. Biol. 256, 173–186 (2003). 12654300 10.1016/s0012-1606(03)00014-9

[37] T. Kinnunen, Z. Huang, J. Townsend, M. M. Gatdula, J. R. Brown, J. D. Esko, J. E. Turnbull, Heparan 2-O-sulfotransferase, hst-2, is essential for normal cell migration in *Caenorhabditis elegans*. Proc. Natl. Acad. Sci. U.S.A. 102, 1507–1512 (2005). 15671174 10.1073/pnas.0401591102 PMC547812

[38] T. T. K. Vuong-Brender, X. Yang, M. Labouesse, *C. elegans* embryonic morphogenesis. Curr. Top. Dev. Biol. 116, 597–616 (2016). 26970644 10.1016/bs.ctdb.2015.11.012

[39] J. M. Bosher, B.-S. Hahn, R. Legouis, S. Sookhareea, R. M. Weimer, A. Gansmuller, A. D. Chisholm, A. M. Rose, J.-L. Bessereau, M. Labouesse, The *Caenorhabditis elegans* vab-10 spectraplakin isoforms protect the epidermis against internal and external forces. J. Cell Biol. 161, 757–768 (2003). 12756232 10.1083/jcb.200302151 PMC2199363

[40] H. Zahreddine, H. Zhang, M. Diogon, Y. Nagamatsu, M. Labouesse, CRT-1/calreticulin and the E3 ligase EEL-1/HUWE1 control hemidesmosome maturation in *C. elegans* development. Curr. Biol. 20, 322–327 (2010). 20153198 10.1016/j.cub.2009.12.061

[41] A. Vasquez-Rifo, G. Jannot, J. Armisen, M. Labouesse, S. I. A. Bukhari, E. L. Rondeau, E. A. Miska, M. J. Simard, Developmental characterization of the microRNA-specific *C. elegans* Argonautes *alg*-1 and *alg*-2. PLOS ONE 7, e33750–e33711 (2012). 22448270 10.1371/journal.pone.0033750 PMC3309000

[42] C. Desai, G. Garriga, S. L. Mclntire, H. R. Horvitz, A genetic pathway for the development of the *Caenorhabditis elegans* HSN motor neurons. Nature 336, 638–646 (1988). 3200316 10.1038/336638a0

[43] C. Rhiner, S. Gysi, E. Fröhli, M. O. Hengartner, A. Hajnal, Syndecan regulates cell migration and axon guidance in *C. elegans*. Development 132, 4621–4633 (2005). 16176946 10.1242/dev.02042

[44] M. E. Pedersen, G. Snieckute, K. Kagias, C. Nehammer, H. A. B. Multhaupt, J. R. Couchman, R. Pocock, An epidermal microRNA regulates neuronal migration through control of the cellular glycosylation state. Science 341, 1404–1408 (2013). 24052309 10.1126/science.1242528

[45] T. K. Kinnunen, Combinatorial roles of heparan sulfate proteoglycans and heparan sulfates in *Caenorhabditis elegans* neural development. PLOS ONE 9, e102919 (2014). 25054285 10.1371/journal.pone.0102919 PMC4108370

[46] K. Saied-Santiago, R. A. Townley, J. D. Attonito, D. S. da Cunha, C. A. Díaz-Balzac, E. Tecle, H. E. Bülow, Coordination of heparan sulfate proteoglycans with Wnt signaling to control cellular migrations and positioning in *Caenorhabditis elegans*. Genetics 206, 1951–1967 (2017). 28576860 10.1534/genetics.116.198739 PMC5560800

[47] Y. Iosilevskii, B. Podbilewicz, Programmed cell fusion in development and homeostasis. Curr. Top. Dev. Biol. 144, 215–244 (2021). 33992154 10.1016/bs.ctdb.2020.12.013

[48] Y. Yang, Y. Zhang, W.-J. Li, Y. Jiang, Z. Zhu, H. Hu, W. Li, J.-W. Wu, Z.-X. Wang, M.-Q. Dong, S. Huang, G. Ou, Spectraplakin induces positive feedback between fusogens and the actin cytoskeleton to promote cell-cell fusion. Dev. Cell 41, 107–120.e4 (2017). 28399395 10.1016/j.devcel.2017.03.006

[49] L. Cochella, O. Hobert, Embryonic priming of a miRNA locus predetermines postmitotic neuronal left/right asymmetry in *C. elegans*. Cell 151, 1229–1242 (2012). 23201143 10.1016/j.cell.2012.10.049 PMC3529140

[50] B. T. Kasper, S. Koppolu, L. K. Mahal, Insights into miRNA regulation of the human glycome. Biochem. Biophys. Res. Commun. 445, 774–779 (2014). 24463102 10.1016/j.bbrc.2014.01.034 PMC4015186

[51] D. M. Kasper, J. Hintzen, Y. Wu, J. J. Ghersi, H. K. Mandl, K. E. Salinas, W. Armero, Z. He, Y. Sheng, Y. Xie, D. W. Heindel, E. J. Park, W. C. Sessa, L. K. Mahal, C. Lebrilla, K. K. Hirschi, S. Nicoli, The N-glycome regulates the endothelial-to-hematopoietic transition. Science 370, 1186–1191 (2020). 33273096 10.1126/science.aaz2121 PMC8312266

[52] A. Sowińska-Seidler, M. Piwecka, E. Olech, M. Socha, A. Latos-Bieleńska, A. Jamsheer, Hyperosmia, ectrodactyly, mild intellectual disability, and other defects in a male patient with an X-linked partial microduplication and overexpression of the KAL1 gene. J. Appl. Genet. 56, 177–184 (2015). 25339597 10.1007/s13353-014-0252-7 PMC4412513

[53] A. J. Giraldez, R. M. Cinalli, M. E. Glasner, A. J. Enright, J. M. Thomson, S. Baskerville, S. M. Hammond, D. P. Bartel, A. F. Schier, MicroRNAs regulate brain morphogenesis in zebrafish. Science 308, 833–838 (2005). 15774722 10.1126/science.1109020

[54] R. J. Parchem, N. Moore, J. L. Fish, J. G. Parchem, T. T. Braga, A. Shenoy, M. C. Oldham, J. L. R. Rubenstein, R. A. Schneider, R. Blelloch, miR-302 is required for timing of neural differentiation, neural tube closure, and embryonic viability. Cell Rep. 12, 760–773 (2015). 26212322 10.1016/j.celrep.2015.06.074 PMC4741278

[55] A. L. Abbott, E. Alvarez-Saavedra, E. A. Miska, N. C. Lau, D. P. Bartel, H. R. Horvitz, V. Ambros, The let-7 microRNA family members mir-48, mir-84, and mir-241 function together to regulate developmental timing in *Caenorhabditis elegans*. Dev. Cell 9, 403–414 (2005). 16139228 10.1016/j.devcel.2005.07.009 PMC3969732

[56] S. Kataruka, M. Modrak, V. Kinterova, R. Malik, D. M. Zeitler, F. Horvat, J. Kanka, G. Meister, P. Svoboda, MicroRNA dilution during oocyte growth disables the microRNA pathway in mammalian oocytes. Nucleic Acids Res. 48, 8050–8062 (2020). 32609824 10.1093/nar/gkaa543 PMC7430632

[57] M. N. McCall, M.-S. Kim, M. Adil, A. H. Patil, Y. Lu, C. J. Mitchell, P. Leal-Rojas, J. Xu, M. Kumar, V. L. Dawson, T. M. Dawson, A. S. Baras, A. Z. Rosenberg, D. E. Arking, K. H. Burns, A. Pandey, M. K. Halushka, Toward the human cellular microRNAome. Genome Res. 27, 1769–1781 (2017). 28877962 10.1101/gr.222067.117 PMC5630040

[58] S. Brenner, The genetics of *Caenorhabditis elegans*. Genetics 77, 71–94 (1974). 4366476 10.1093/genetics/77.1.71 PMC1213120

[59] A. Paix, A. Folkmann, D. Rasoloson, G. Seydoux, High efficiency, homology-directed genome editing in *Caenorhabditis elegans* using CRISPR-Cas9 ribonucleoprotein complexes. Genetics 201, 47–54 (2015). 26187122 10.1534/genetics.115.179382 PMC4566275

[60] S. R. Sternberg, Biomedical image processing. Computertomographie 16, 22–34 (1983).

[61] T. Smith, A. Heger, I. Sudbery, UMI-tools: Modeling sequencing errors in unique molecular identifiers to improve quantification accuracy. Genome Res. 27, 491–499 (2017). 28100584 10.1101/gr.209601.116 PMC5340976

[62] A. Dobin, C. A. Davis, F. Schlesinger, J. Drenkow, C. Zaleski, S. Jha, P. Batut, M. Chaisson, T. R. Gingeras, STAR: Ultrafast universal RNA-seq aligner. Bioinformatics 29, 15–21 (2013). 23104886 10.1093/bioinformatics/bts635 PMC3530905

[63] S. Anders, P. T. Pyl, W. Huber, HTSeq—A Python framework to work with high-throughput sequencing data. Bioinformatics 31, 166–169 (2015). 25260700 10.1093/bioinformatics/btu638 PMC4287950

[64] M. I. Love, W. Huber, S. Anders, Moderated estimation of fold change and dispersion for RNA-seq data with DESeq2. Genome Biol. 15, 550 (2014). 25516281 10.1186/s13059-014-0550-8 PMC4302049

[65] S. G. Clark, C. Chiu, *C. elegans* ZAG-1, a Zn-finger-homeodomain protein, regulates axonal development and neuronal differentiation. Development 130, 3781–3794 (2003). 12835394 10.1242/dev.00571

[66] D. Didiano, O. Hobert, Perfect seed pairing is not a generally reliable predictor for miRNA-target interactions. Nat. Struct. Mol. Biol. 13, 849–851 (2006). 16921378 10.1038/nsmb1138

