# Supplementary material for: An ancient and essential miRNA family controls cellular interaction pathways in C. elegans
Source: Sci Adv. 2025 Sep 3;11(36):eadz1934. doi: 10.1126/sciadv.adz1934 (PMC12407057; doi:10.1126/sciadv.adz1934)
Supplement: Supplementary file 1 — Figs. S1 to S6 Table S1 Legends for movies S1 and S2 Legends for data S1 and S2 References [file sciadv.adz1934_sm.pdf]

Science Advances
AAAS

Supplementary Materials for

An ancient and essential miRNA family controls cellular interaction pathways in *C. elegans*

Emilio M. Santillan *et al.*

Corresponding author: Luisa Cochella, mcochel1@jhmi.edu

Sci. Adv. **11**, eadz1934 (2025)
DOI: 10.1126/sciadv.adz1934

The PDF file includes:

Figs. S1 to S6
Table S1
Legends for movies S1 and S2
Legends for data S1 and S2
References

Other Supplementary Material for this manuscript includes the following:

Movies S1 and S2
Data S1 and S2

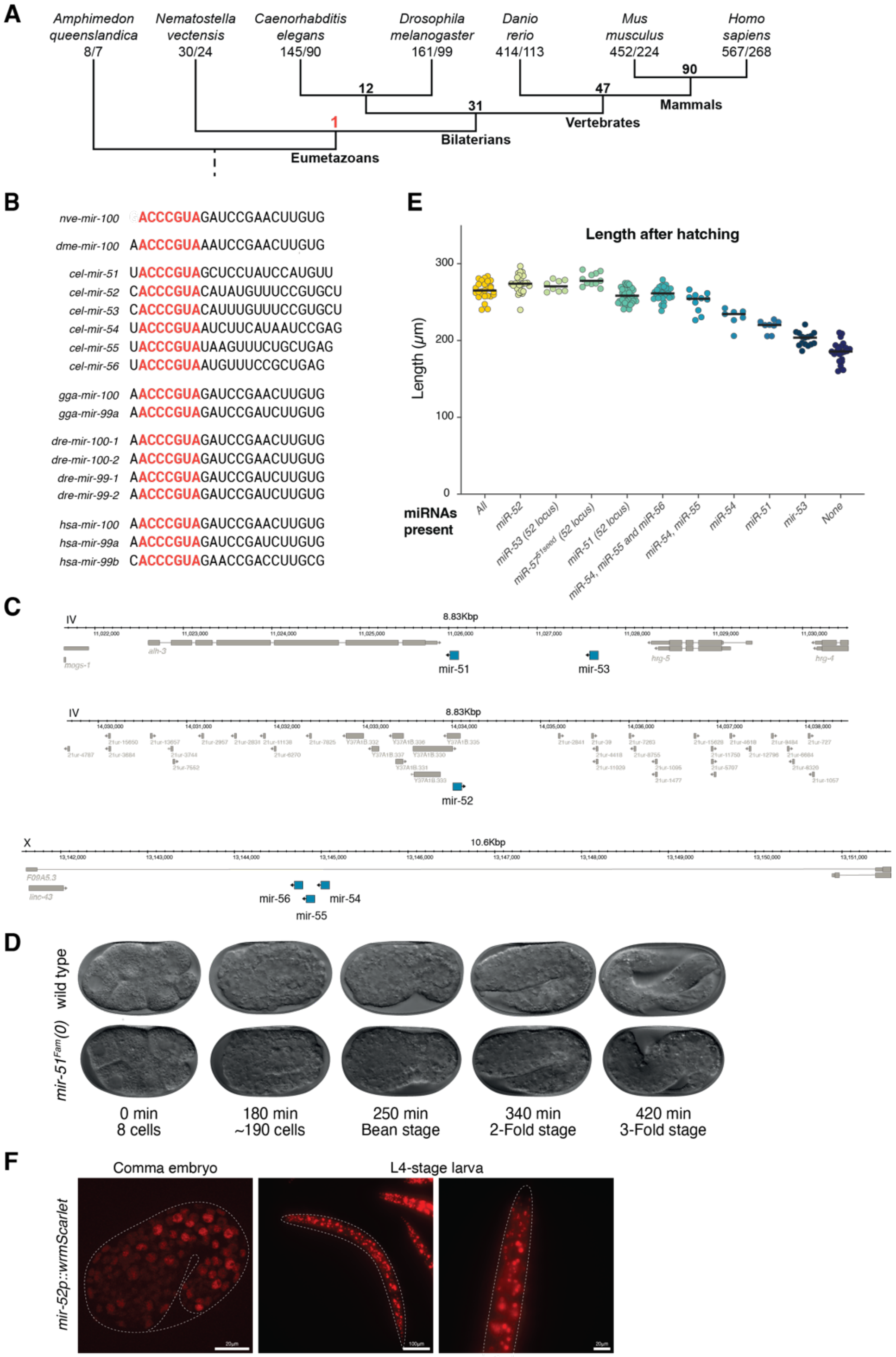

Fig. S1. The miR-51^{fam} is conserved and necessary for development. A. Simplified phylogenetic tree highlighting the number of conserved miRNA families across selected clades (in bold). Also shown are the numbers of miRNA/number of miRNA families for representative species. miR-100 is the sole miRNA family conserved across Eumetazoa (in red). Data from (*5*). **B.** Alignment of miR-100 family members from *Nematostella vectensis (nve), Drosophila melanogaster (dme), Caenorhabditis elegans (cel), Gallus gallus (gga), Danio rerio (dre)* and *Homo sapiens (hsa)*. Seed sequences are highlighted in red. **C.** Genomic location of the four miR-51 family loci in *C. elegans.* miR-54, -55, -56 are present in a cluster expressed from a single transcript, the other three family members each have independent promoters. **D.** Representative images of developing *C. elegans* embryos with or without miR-51 family miRNAs, showing that defects begin during morphogenesis. The complete family null contains three deletion alleles: *nDf67, n4100 IV; nDf58 X* (*8*). **E.** Length of L1s with different dosage of the miR-51^{Fam}, or corresponding to the replacement experiments shown in Fig. 1C. **F.** Representative images of embryo and larvae with wrmScarlet insertion in the miR-52 locus. The miR-52 hairpin was replaced by a worm codon optimized mScarlet using CRISPR/Cas9.

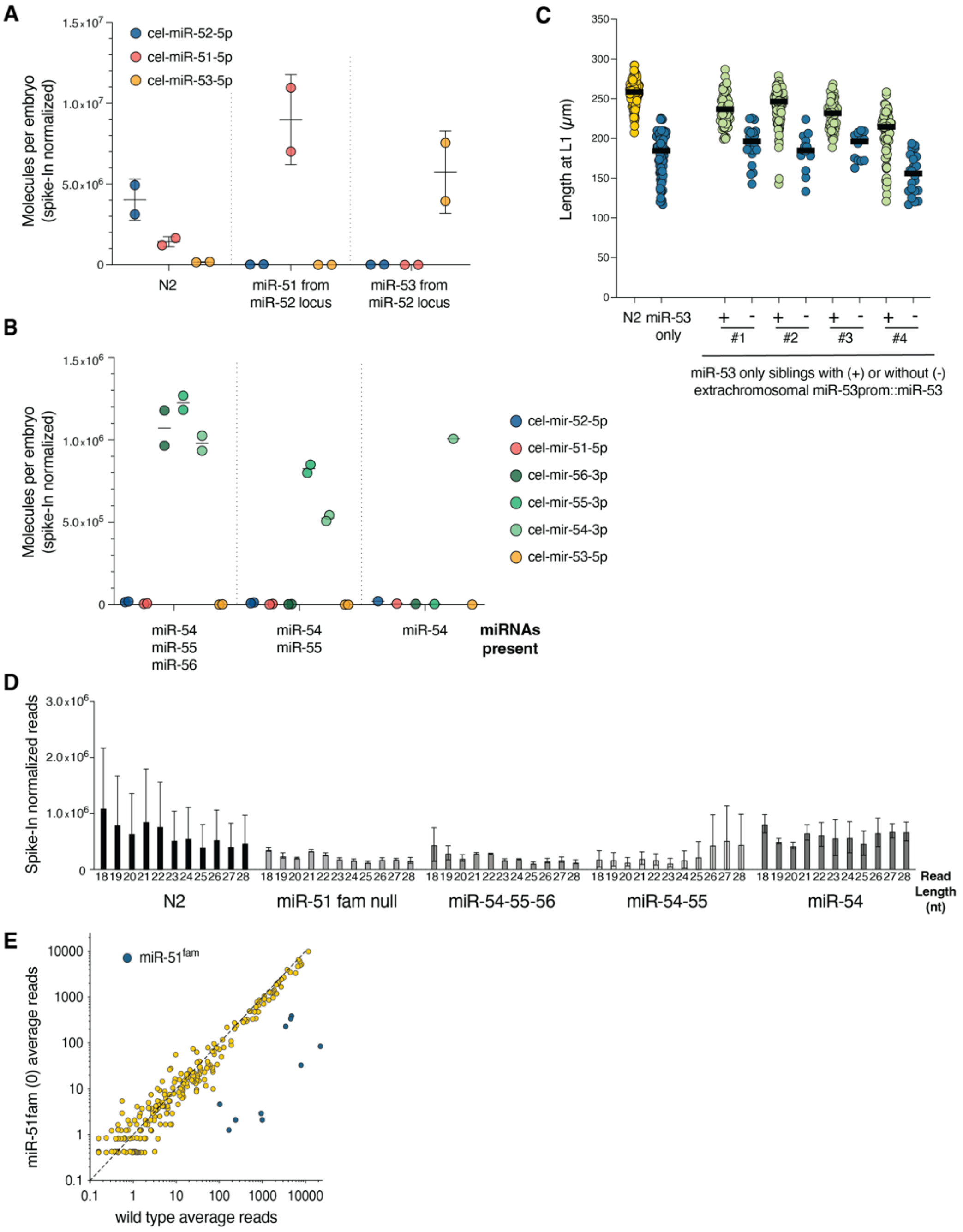

Fig. S2. miR-51 family members are differentially expressed and function in a dose-dependent manner. A. Absolute quantification of selected miR-51 family members by small RNA sequencing using spike-ins, in embryos in which the miR-52 hairpin was replaced by that of miR-51 or miR-53, compared to wild type. **B.** Absolute quantification of all miR-51 family members as in A, in embryos with progressive deletions of the miR-54 cluster, and all other miR-51family loci deleted. **C.** Length measurements for larvae with only miR-53 (blue) compared to siblings carrying a multi-copy extrachromosomal element expressing additional miR-53 (green). Four independent extrachromosomal transgenic lines are shown. **D.** Number of spike-in normalized reads of the indicated lengths (18-28 nt) that do not map to microRNAs or piRNAs, in embryos lacking different miR-51 family members. **E.** Comparison of miRNA abundances in wild type and miR-51family null embryos. Most miRNAs are unchanged (yellow) while the miR-51 family (blue) is depleted, as expected.

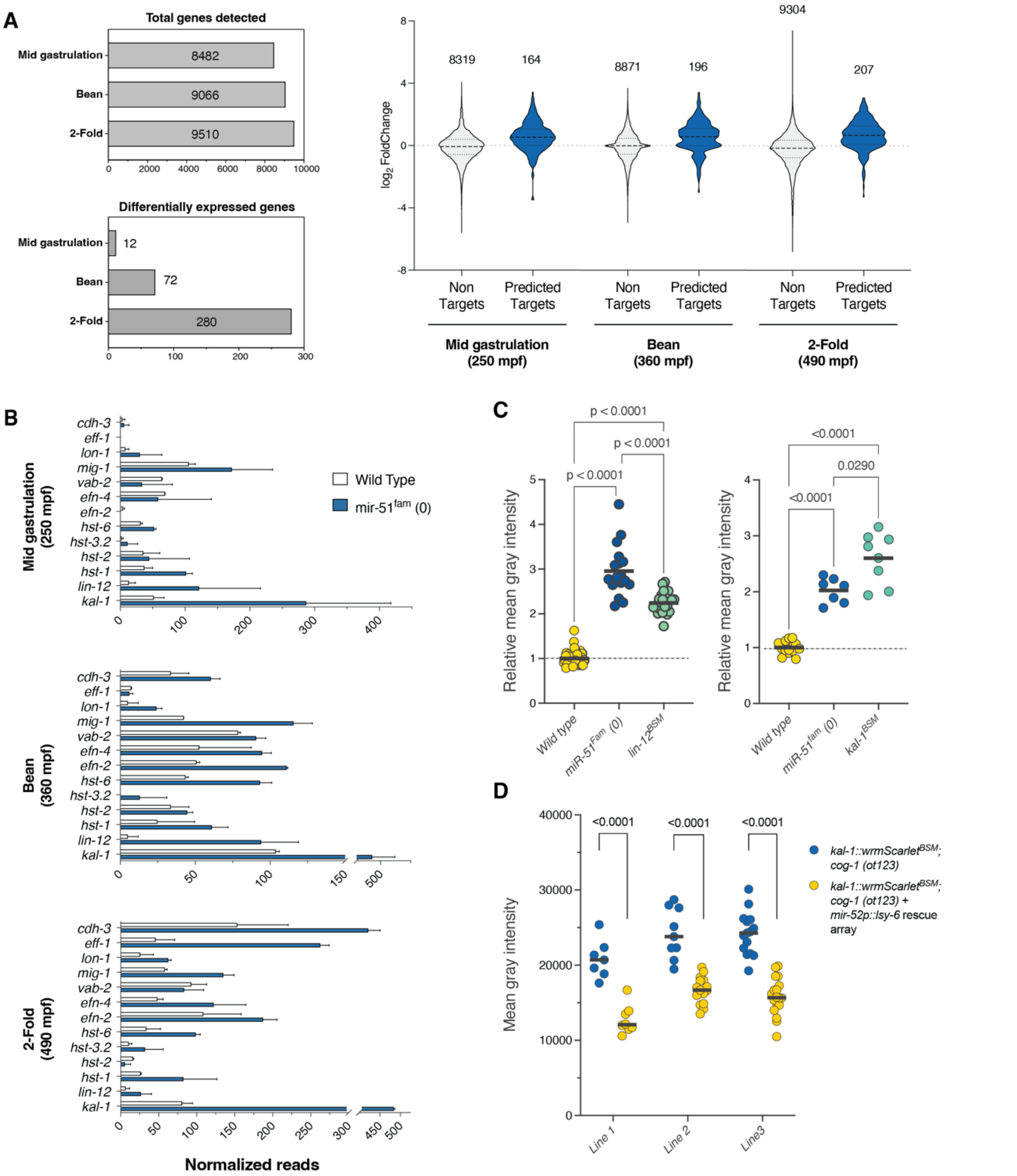

Fig. S3. Identification of miR-51 family targets. A. General description of the mRNA sequencing data at different timepoints of embryogenesis comparing wild type and miR-5^{Fam} null embryos. mpf = minutes post fertilization. **B.** Average normalized reads for selected targets of miR-51^{Fam} in wild type and miR-5^{Fam} null embryos. **C.** Fluorescence quantification in animals with endogenously tagged *lin-12*/Notch (left) or *kal-1*/Anosmin1 (right) loci. Genes were tagged by inserting a T2A::wrmScarlet::H2B cassette before the stop codon (analogous to the tagging of *hst-3.2* in Fig. 2D). Fluorescence was measured in whole embryos (*kal-1*) or L1 larvae (*lin-12*) in wild type background, or upon deletion of miR-51family, or upon mutation of the miR-51 binding sites in the 3' UTR (BSM). **D.** Fluorescence quantification of the endogenous KAL-1::T2A::Scarlet::H2B::miR-51 binding site mutant (BSM) in embryos with or without an extrachromosomal element expressing the miRNA *lsy-6* under the miR-52 promoter. *lsy-6* binds the BSM in *kal-1* restoring repression. *cog-1 (ot123)*, is a mutation in the 3' UTR of *cog-1*, the functional target of *lsy-6*, to avoid ectopic silencing in cells that normally do not express *lsy-6.* This mutation does not cause any phenotypes except the loss of functional asymmetry in a specific gustatory neuron pair.

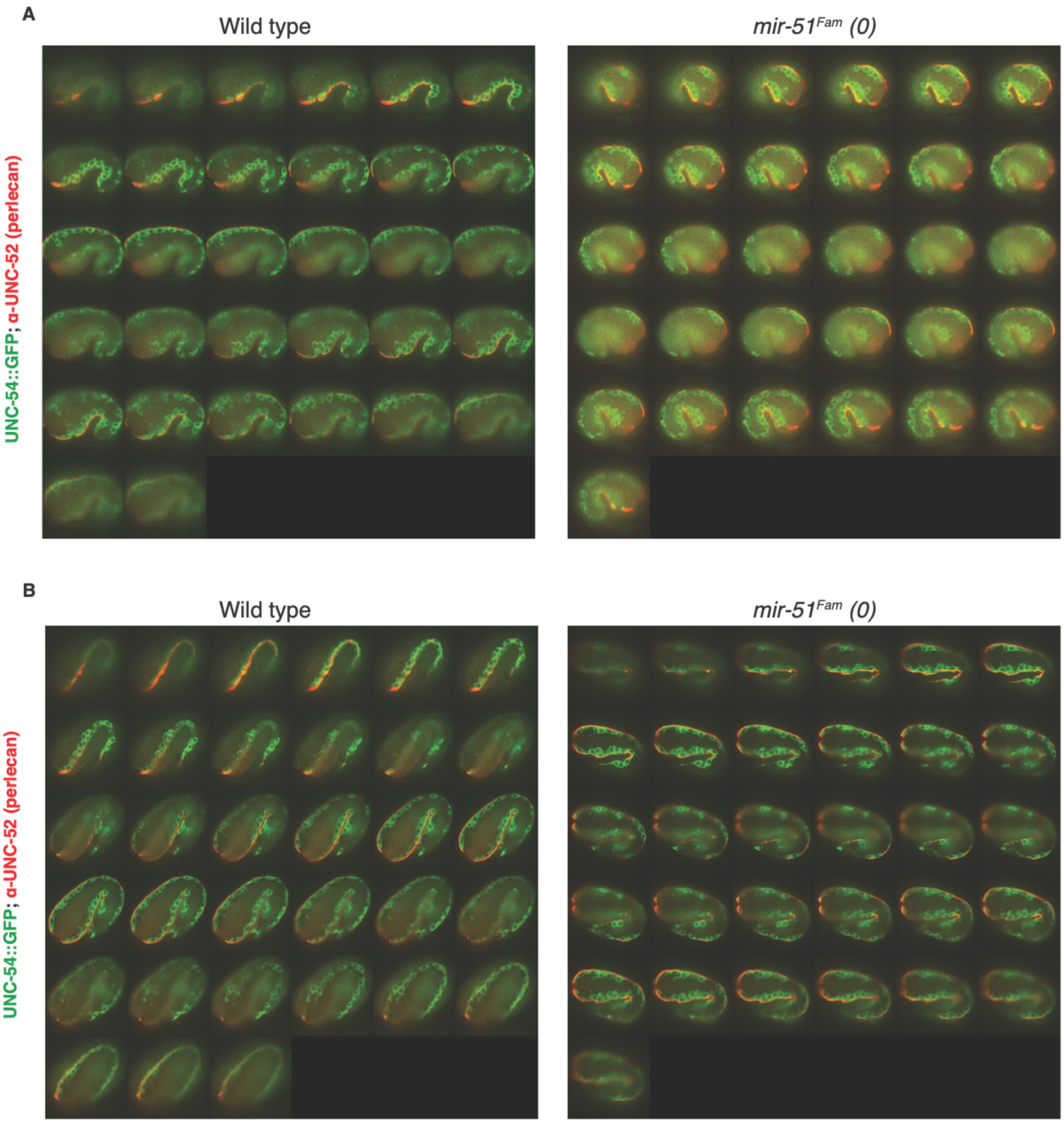

Fig. S4. Muscle development appears normal in animals lacking the miR-51 family. A. Representative images of late comma/1.5-fold stage embryos showing immunofluorescence staining for perlecan (UNC-52, red) and muscle cells marked by an endogenous myosin heavy chain (UNC-54::GFP, green). In wild-type embryos, perlecan is deposited in four longitudinal basal membrane tracks underlying the muscle cell bundles. Shown are multiple z-planes per embryo to capture all bundles. Mutant embryos exhibit a similar pattern of perlecan basal membrane deposition underlying muscle cells. **B.** At the 2-fold stage, the characteristic basal membrane pattern of perlecan (red) underlying UNC-54::GFP-labeled muscle cells (green) is also observed in both wild-type and mutant embryos.

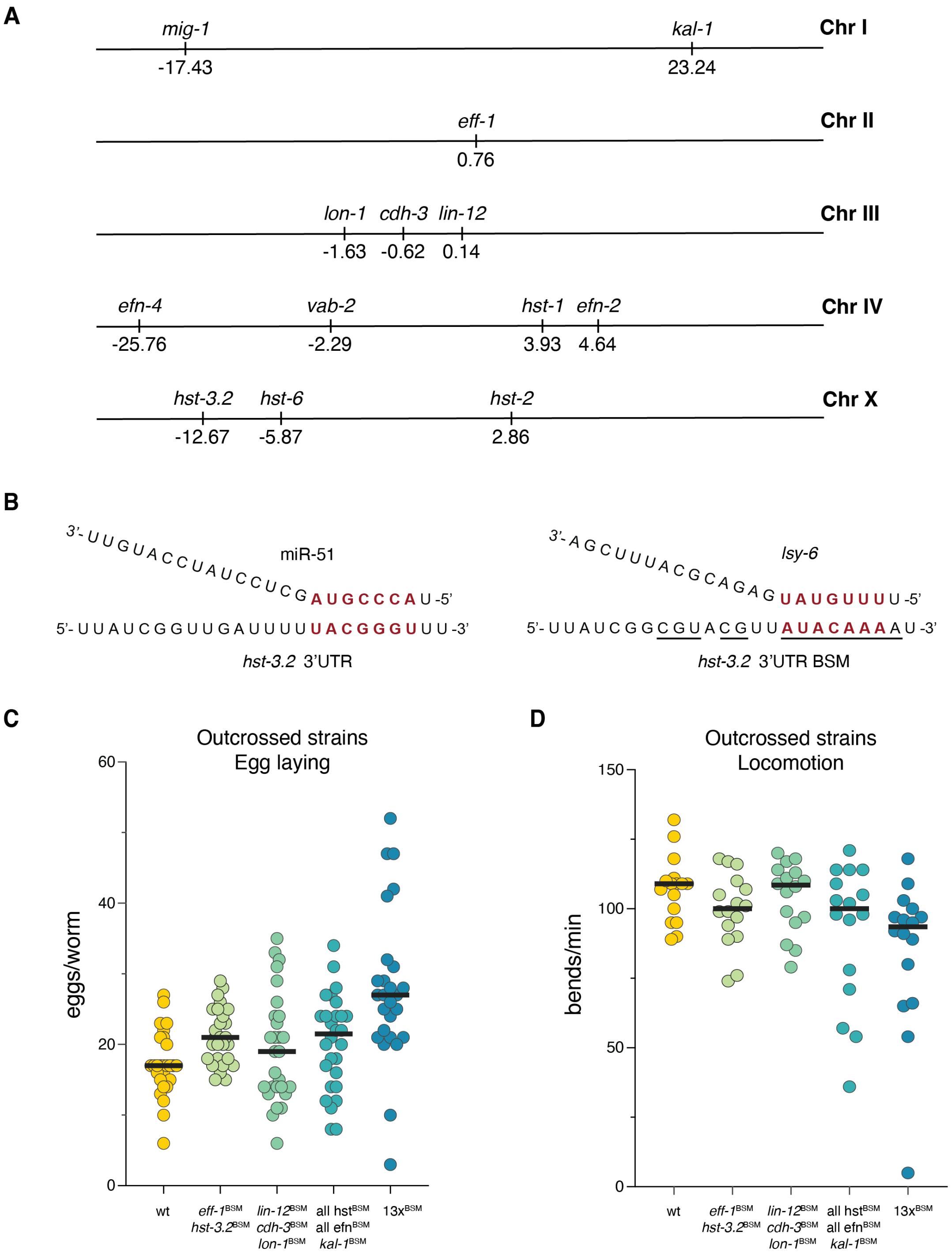

Fig. S5. miR-51 binding site mutation design and effect of loss of miR-51 binding in animals with various target mutations. A. Relative genetic distances of the targets of miR-51^{Fam} over various *C. elegans* chromosomes (no targets are present on Chr V). **B.** (Left) Schematic of the wild type pairing between *hst-3.2* 3' UTR and miR-51, with the seed sequence highlighted in red. (Right) Mutated binding site in *hst-3.2* that enables pairing with *lsy-6* (*66*). Analogous mutations were inserted in all thirteen targets shown in A. **C.** Egg retention measured in strains obtained by outcrossing the 13xBSM strain to wild type (N2) or to the 8xBSM strain, to obtain new strains with different allele combinations. We obtained a 2xBSM strain with mutations in *eff-1* and *hst-3.2* binding sites, a 3xBSM with only the Chr III mutants (*lin-12, cdh-3, lon-1*), and an 8xBSM with all ephrins, HSTs and *kal-1* mutated. **D.** Locomotion measured in the same strains shown in C.

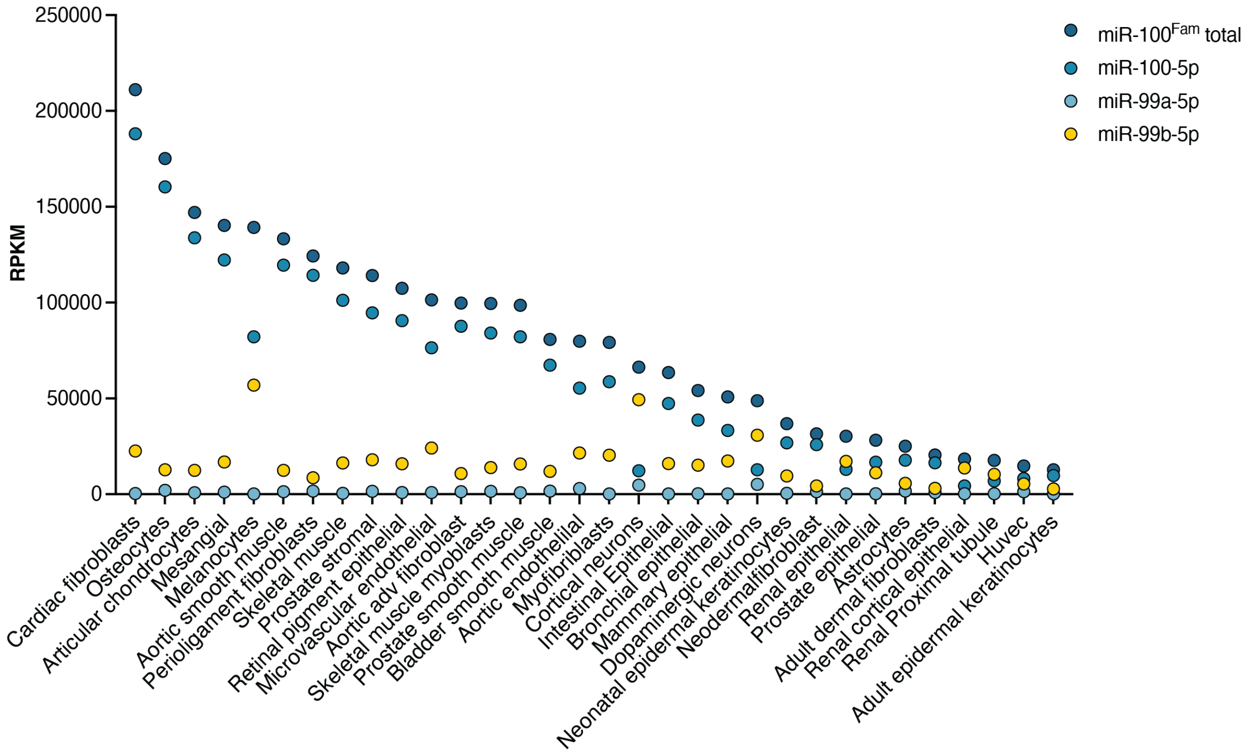

Fig. S6. Relative miRNA expression for all members of the miR-100 family in humans across a panel of primary cell types and tissues. Point in dark blue represents the sum of all three family members. Original data from (*57*).

Table S1.
List of *C. elegans* strains used in this study.

Strain	Genotype	Comment	Reference
N2	*wild type*	Wild isolate from Bristol. Given to S. Brenner ca. 1966.	
MLC1800	*nDf67, n4100 IV; nDf58 X; lucEx1057*	miR-51Fam (0); lucEx1057: exarray expressing fosmid containing miR-54C. Markers: ttx-3::gfp + mir-35p::gfp	This study.
MT17136	*nDf67 IV; nDf58 X*	Only expresses miR-52 from the miR-51Fam	(*8*)
MLC2290	*nDf67, n4100 IV*	Only expresses the miR-54C from the miR-51Fam	This study.
MT17446	*n4113, n4100 IV; nDf58 X*	Only expresses miR-51 from the miR-51Fam. Slow growing	(*8*)
MLC2336	*n4473, n4100 / tmC9 IV; nDf58 X*	Only expresses miR-53 from the miR-51Fam. Balanced with tmC9 from Mishima lab.	This study.
MLC1997	*nDf67, mir-52 (luc143); nDf58 X*	luc143: pre-mir-52 hairpin endogenously replaced by pre-mir-51 hairpin with CRISPR.	This study.
MLC1917	*nDf67, mir-52 (luc140); nDf58 X*	luc143: pre-mir-52 hairpin endogenously replaced by pre-mir-53 hairpin with CRISPR.	This study.
MLC1918	*nDf67, mir-52 (luc141); nDf58 X*	luc143: pre-mir-52 hairpin endogenously replaced by pre-mir-57 (with seed mutation) hairpin with CRISPR.	This study.
MLC2058	*nDf67, mir-52 (luc151); nDf58 X; lucEx1057*	luc151: pre-mir-52 hairpin endogenously replaced by pre-mir-57 hairpin with CRISPR. lucEx1057: fosmid expressing miR-54C. Markers: ttx-3::gfp + mir-35p::gfp	This study.
MLC2237	*mir-51 (luc163)*	luc162: replacement of pre-mir-51 hairpin by a wrmScarlet::TY1::H2B::tbb-2-3'UTR cassette.	This study.
MLC2238	*mir-53 (luc164)*	luc164: replacement of pre-mir-53 hairpin by a wrmScarlet::TY1::H2B::tbb-2-3'UTR cassette.	This study.
MLC2280	*miR-52 (luc173)*	luc173: replacement of pre-mir-52 hairpin by a wrmScarlet::TY1::H2B::tbb-2-3'UTR cassette.	This study.
MLC2394	*n4100, nDf67 IV; mir-56 (luc177) X*	Only expresses miR-54 and miR-55 from miR-51Fam. Luc177: clean deletion of the pre-miR-56 hairpin by CRISPR.	This study.
MLC2393	*n4100, nDf67 IV; miR-55-56 (luc180) X*	Only expresses miR-54 (slow growing) from miR-51Fam. Luc180: clean deletion pre-miR-56 and pre-mir-55 hairpins by CRISPR.	This study.
GOU2043	*vab-10a (cas602[vab10a::gfp]) I*	cas602: GFP inserted into the endogenous vab-10a gene at its C-terminus by CRISPR. miR-51Fam (0); lucEx1057: Ex array	(*48*)

		expressing fosmid containing miR-54C. Markers: ttx-3::gfp + mir-35p::gfp	
MLC2676	*vab-10a (cas602[vab10a::gfp]) I; nDf67, n4100 IV; nDf58 X; lucEx1057*	cas602: GFP inserted into the endogenous vab-10a gene at its C-terminus by CRISPR. miR-51Fam (0); lucEx1057: Ex array expressing fosmid containing miR-54C. Markers: ttx-3::gfp + mir-35p::gfp	This study.
MLC2654	*zdIs13 [tph-1::GFP] IV*	Transcriptional tph-1::gfp reporter. From SK4013, outcrossed 2x to N2. zdIs13: insertion of a tph-1::gfp expressing array (marks serotonerginc neurons, including HSN)	This study; (*65*)
MLC2662	*zdIs13 [tph-1::GFP] , n4100; mir-55-56 (luc180) IV*	Expresses only miR-51, miR-53 and miR-54. Transcriptional tph-1::gfp reporter from SK4013, outcrossed 2x to N2. zdIs13: insertion of a tph-1::gfp expressing array (marks serotonerginc neurons, including HSN). Luc180: clean deletion pre-miR-56 and pre-mir-55 hairpins by CRISPR.	This study; (*65*)
MLC2793	*lucEx1388*	lucEx1388: simple ex-array expressing unc-122::dsRED.	This study.
MLC2748	*n4100, nDf67 IV; miR-55-56 (luc180) X; lucEx1385*	Only expresses miR-54 (slow growing) from miR-51Fam. Luc180: clean deletion pre-miR-56 and pre-mir-55 hairpins by CRISPR. lucEx1385: simple ex-Array expressing unc-122::dsRED (marks coelomocytes). Line 1	This study.
MLC2749	*n4100, nDf67 IV; miR-55-56 (luc180) X; lucEx1386*	Only expresses miR-54 (slow growing) from miR-51Fam. Luc180: clean deletion pre-miR-56 and pre-mir-55 hairpins by CRISPR. lucEx1386: simple ex-Array expressing unc-122::dsRED (marks coelomocytes). Line 2	This study.
MLC2750	*n4100, nDf67 IV; miR-55-56 (luc180) X; lucEx1387*	Only expresses miR-54 (slow growing) from miR-51Fam. Luc180: clean deletion pre-miR-56 and pre-mir-55 hairpins by CRISPR. lucEx1385: simple ex-Array expressing unc-122::dsRED (marks coelomocytes). Line 3	This study.
MLC2255	*kal-1 (luc158) I.*	luc158: cassette containing T2A::wrmScarlet::TY1::H2B inserted in the stop codon position of kal-1.	This study.
MLC2236	*kal-1 (luc158) I; nDf67,n4100 IV; nDf58 X; lucEx1057*	miR-51Fam (0); luc158: cassette containing T2A::wrmScarlet::TY1::H2B inserted in the stop codon position of kal-1; lucEx1057: Ex array expressing fosmid containing miR-54C. Markers: ttx-3::gfp + mir-35p::gfp	This study.

MLC2395	*kal-1 (luc158) I; n4100, nDf67 IV; miR-55-56 (luc180) X*	Only expresses miR-54 (slow growing) from miR-51Fam. Luc180: clean deletion pre-miR-56 and pre-mir-55 hairpins by CRISPR. luc158: cassette containing T2A::wrmScarlet::TY1::H2B inserted in the stop codon position of kal-1.	This study.
MLC2396	*kal-1 (luc158) I; n4100, nDf67 IV; mir-56 (luc177) X*	Only expresses miR-54 and miR-55 from miR-51Fam. Luc177: clean deletion of the pre-miR-56 hairpin by CRISPR. luc158: cassette containing T2A::wrmScarlet::TY1::H2B inserted in the stop codon position of kal-1.	This study.
MLC2684	*kal-1 (luc158) I; nDf67, n4100 IV*	Only expresses the miR-54C from the miR-51Fam. luc158: cassette containing T2A::wrmScarlet::TY1::H2B inserted in the stop codon position of kal-1.	This study.
MLC2441	*kal-1 (luc158, luc186) I.*	luc158: cassette containing T2A::wrmScarlet::TY1::H2B inserted in the stop codon position of kal-1. luc186: miR-51Fam seed recognition element replaced by the lsy-6 complete binding site sequence (*66*).	This study.
MLC2469	*hst-3.2 (luc190) X.*	luc190: cassette containing T2A::mNeonGreen::TY1::H2B inserted in the stop codon position of hst-3.2.	This study.
MLC2542	*nDf67, n4100 IV; nDf58, hst-3.2 (luc190) X; lucEx1057*	miR-51Fam (0); luc190: cassette containing T2A::mNeonGreen::TY1::H2B inserted in the stop codon position of hst-3.2.; lucEx1057: exarray expressing fosmid containing miR-54C. Markers: ttx-3::gfp + mir-35p::gfp	This study.
MLC2521	*n4100, nDf67 IV; hst-3.2 (luc190) X.*	Only expresses the miR-54C from the miR-51Fam. luc190: cassette containing T2A::mNeonGreen::TY1::H2B inserted in the stop codon position of hst-3.2.	This study.
MLC2541	*n4100, nDf67 IV; mir-56 (luc177), hst-3.2 (luc190) X*	Only expresses miR-54 and miR-55 from miR-51Fam. luc177: clean deletion of the pre-miR-56 hairpin by CRISPR. luc190: cassette containing T2A::mNeonGreen::TY1::H2B inserted in the stop codon position of hst-3.2.	This study.
MLC2548	*n4100, nDf67 IV; miR-55-56 (luc180), hst-3.2 (luc190) X*	Only expresses miR-54 (slow growing) from miR-51Fam. Luc180: clean deletion pre-miR-56 and pre-mir-55 hairpins by CRISPR. luc190: cassette containing T2A::mNeonGreen::TY1::H2B inserted in the stop codon position of hst-3.2.	This study.
MLC2573	*hst-3.2 (luc190, luc199) X.*	luc190: cassette containing T2A::mNeonGreen::TY1::H2B inserted in the stop codon position of hst-3.2. luc199: miR-51Fam seed recognition element replaced by the lsy-6 complete binding site	This study.

		sequence (*66*), modified to include a BsiWI restriction site (CGtacgTTATACAAAA).	
MLC2575	*lin-12 (luc200) III.*	luc200: cassette containing T2A::wrmScarlet::TY1::H2B inserted in the stop codon position of lin-12.	This study.
MLC2576	*lin-12 (luc200) III; n4100, nDf67 IV*	Only expresses the miR-54C from the miR-51Fam. luc200: cassette containing T2A::wrmScarlet::TY1::H2B inserted in the stop codon position of lin-12.	This study.
MLC2577	*lin-12 (luc200) III; n4100, nDf67 IV; mir-56 (luc177) X.*	Only expresses miR-54 and miR-55 from miR-51Fam. luc177: clean deletion of the pre-miR-56 hairpin by CRISPR. luc200: cassette containing T2A::wrmScarlet::TY1::H2B inserted in the stop codon position of lin-12.	This study.
MLC2578	*lin-12 (luc200) III; n4100, nDf67 IV; miR-55-56 (luc180) X.*	Only expresses miR-54 (slow growing) from miR-51Fam. Luc180: clean deletion pre-miR-56 and pre-mir-55 hairpins by CRISPR. luc200: cassette containing T2A::wrmScarlet::TY1::H2B inserted in the stop codon position of lin-12.	This study.
MLC2579	*lin-12 (luc200) III; nDf67, n4100 IV; nDf58 X; lucEx1057*	miR-51Fam (0); luc200: cassette containing T2A::wrmScarlet::TY1::H2B inserted in the stop codon position of lin-12.; lucEx1057: Exarray expressing fosmid containing miR-54C. Markers: ttx-3::gfp + mir-35p::gfp	This study.
MLC2574	*lin-12 (luc198) III.*	luc198: cassette containing T2A::wrmScarlet::TY1::H2B inserted in the stop codon position of lin-12. Both miR-51Fam seed recognition elements replaced by the lsy-6 complete binding site sequence (*66*)	This study.
MLC2646	*kal-1 (luc216) I; hst-3.2 (luc215), hst-6 (luc218) X*	luc215-218: miR-51Fam seed recognition element replaced for the lsy-6 binding site (*66*), modified to include a BsiWI restriction site (CGtacgTTATACAAAA).	This study.
MLC2703	*efn-4 (luc242), vab-2 (luc244), efn-2 (luc246) IV*	luc242-246: miR-51Fam seed recognition element replaced for the lsy-6 binding site (*66*), modified to include a BsiWI restriction site (CGtacgTTATACAAAA).	This study.
MLC2745	*kal-1 (luc216) I; efn-4 (luc242), vab-2 (luc244), efn-2 (luc246) IV; hst-3.2 (luc215), hst-6 (luc218) X*	luc215-246: miR-51Fam seed recognition element replaced for the lsy-6 binding site (*66*), modified to include a BsiWI restriction site (CGtacgTTATACAAAA).	This study.

MLC2772	*kal-1 (luc216) I; eff-1 (luc259) II; efn-4 (luc242), vab-2 (luc244), efn-2 (luc246) IV; hst-3.2 (luc215), hst-6 (luc218) X*	luc215-259: miR-51Fam seed recognition element replaced for the lsy-6 binding site (*66*), modified to include a BsiWI restriction site (CGtacgTTATACAAAA).	This study.
MLC2774	*kal-1 (luc216) I; eff-1 (luc259) II; efn-4 (luc242), vab-2 (luc244), hst-1 (luc261), efn-2 (luc246) IV; hst-3.2 (luc215), hst-6 (luc218) X*	luc215-261: miR-51Fam seed recognition element replaced for the lsy-6 binding site (*66*), modified to include a BsiWI restriction site (CGtacgTTATACAAAA).	This study.
MLC2778	*kal-1 (luc216) I; eff-1 (luc259) II; efn-4 (luc242), vab-2 (luc244), hst-1 (luc261), efn-2 (luc246) IV; hst-3.2 (luc215), hst-6 (luc218), hst-2 (luc263) X*	luc215-263: miR-51Fam seed recognition element replaced for the lsy-6 binding site (*66*), modified to include a BsiWI restriction site (CGtacgTTATACAAAA).	This study.
MLC2791	*kal-1 (luc216) I; eff-1 (luc259) II; lon-1 (luc268) III; efn-4 (luc242), vab-2 (luc244), hst-1 (luc261), efn-2 (luc246) IV; hst-3.2 (luc215), hst-6 (luc218), hst-2 (luc263) X*	luc215-268: miR-51Fam seed recognition element replaced for the lsy-6 binding site (*66*), modified to include a BsiWI restriction site (CGtacgTTATACAAAA).	This study.
MLC2805	*kal-1 (luc216) I; eff-1 (luc259) II; lon-1 (luc268), lin-12 (luc271) III; efn-4 (luc242), vab-2 (luc244), hst-1 (luc261), efn-2 (luc246) IV; hst-3.2 (luc215), hst-6 (luc218), hst-2 (luc263) X*	luc215-271: miR-51Fam seed recognition element replaced for the lsy-6 binding site (*66*), modified to include a BsiWI restriction site (CGtacgTTATACAAAA).	This study.
MLC2828	*mig-1 (luc272), kal-1 (luc216) I; eff-1 (luc259) II; lon-1 (luc268), lin-12 (luc271) III; efn-4 (luc242), vab-2 (luc244), hst-1 (luc261), efn-2*	luc215-272: miR-51Fam seed recognition element replaced for the lsy-6 binding site (*66*), modified to include a BsiWI restriction site (CGtacgTTATACAAAA).	This study.

	(luc246) IV; hst-3.2 (luc215), hst-6 (luc218), hst-2 (luc263) X		
MLC2835	*mig-1 (luc272), kal-1 (luc216) I; eff-1 (luc259) II; lon-1 (luc268), cdh-3 (luc275) lin-12 (luc271) III; efn-4 (luc242), vab-2 (luc244), hst-1 (luc261), efn-2 (luc246) IV; hst-3.2 (luc215), hst-6 (luc218), hst-2 (luc263) X*	luc215-275: miR-51Fam seed recognition element replaced for the lsy-6 binding site (*66*), modified to include a BsiWI restriction site (CGtacgTTATACAAAA).	This study.
MLC2858	*mig-1 (luc272), cog-1(luc280), kal-1 (luc216) I; eff-1 (luc259) II; lon-1 (luc268), cdh-3 (luc275) lin-12 (luc271) III; efn-4 (luc242), vab-2 (luc244), hst-1 (luc261), efn-2 (luc246) IV; hst-3.2 (luc215), hst-6 (luc218), hst-2 (luc263) X*	luc215-275: miR-51Fam seed recognition element replaced for the lsy-6 binding site (*66*), modified to include a BsiWI restriction site (CGtacgTTATACAAAA). luc280: small deletion that covers both binding sites for lsy-6 in the cog-1 3UTR.	This study.
MLC2936	*mig-1 (luc272), cog-1(luc280), kal-1 (luc216) I; eff-1 (luc259) II; lon-1 (luc268), cdh-3 (luc275) lin-12 (luc271) III; efn-4 (luc242), vab-2 (luc244), hst-1 (luc261), efn-2 (luc246) IV; hst-3.2 (luc215), hst-6 (luc218), hst-2 (luc263) X*	luc215-275: miR-51Fam seed recognition element replaced for the lsy-6 binding site (*66*), modified to include a BsiWI restriction site (CGtacgTTATACAAAA). luc280: small deletion that covers both binding sites for lsy-6 in the cog-1 3UTR. lucEx1417: expresses lsy-6 under the mir-52 promoter. Pick myo-2::mcherry positive worms to mantain.	This study.

MLC2885	*eff-1 (luc259) II; hst-3.2 (luc215) X; lucEx1399 (tph-1p::gfp, ttx-3::mcherry)*	luc215-259: miR-51Fam seed recognition element replaced for the lsy-6 binding site (*66*), modified to include a BsiWI restriction site (CGtacgTTATACAAAA). lucEx1399: tph-1p::gfp, ttx-3::mcherry. Outcrossed isolate 1	This study.
MLC2886	*eff-1 (luc259) II; hst-3.2 (luc215) X; lucEx1399 (tph-1p::gfp, ttx-3::mcherry)*	luc215-259: miR-51Fam seed recognition element replaced for the lsy-6 binding site (*66*), modified to include a BsiWI restriction site (CGtacgTTATACAAAA). lucEx1399: tph-1p::gfp, ttx-3::mcherry. Outcrossed isolate 2	This study.
MLC2887	*lon-1 (luc268), cdh-3 (luc275) lin-12 (luc271) III; lucEx1399 (tph-1p::gfp, ttx-3::mcherry)*	luc268-275: miR-51Fam seed recognition element replaced for the lsy-6 binding site (*66*), modified to include a BsiWI restriction site (CGtacgTTATACAAAA). lucEx1399: tph-1p::gfp, ttx-3::mcherry. Outcrossed isolate 1	This study.
MLC2888	*lon-1 (luc268), cdh-3 (luc275) lin-12 (luc271) III; lucEx1399 (tph-1p::gfp, ttx-3::mcherry)*	luc268-275: miR-51Fam seed recognition element replaced for the lsy-6 binding site (*66*), modified to include a BsiWI restriction site (CGtacgTTATACAAAA). lucEx1399: tph-1p::gfp, ttx-3::mcherry. Outcrossed isolate 2	This study.
MLC2889	*kal-1 (luc216) I; efn-4 (luc242), vab-2 (luc244), hst-1 (luc261), efn-2 (luc246) IV; hst-3.2 (luc215), hst-6 (luc218), hst-2 (luc263) X; lucEx1399 (tph-1::gfp, ttx-3::mcherry*	luc215-263: miR-51Fam seed recognition element replaced for the lsy-6 binding site (*66*), modified to include a BsiWI restriction site (CGtacgTTATACAAAA). lucEx1399: tph-1p::gfp, ttx-3::mcherry. Outcrossed isolate 1	This study.
MLC2890	*kal-1 (luc216) I; efn-4 (luc242), vab-2 (luc244), hst-1 (luc261), efn-2 (luc246) IV; hst-3.2 (luc215), hst-6 (luc218), hst-2 (luc263) X; lucEx1399 (tph-1::gfp, ttx-3::mcherry*	luc215-263: miR-51Fam seed recognition element replaced for the lsy-6 binding site (*66*), modified to include a BsiWI restriction site (CGtacgTTATACAAAA). lucEx1399: tph-1p::gfp, ttx-3::mcherry. Outcrossed isolate 2	This study.

MLC2891	*mig-1 (luc272), kal-1 (luc216) I; eff-1 (luc259) II; lon-1 (luc268), cdh-3 (luc275) lin-12 (luc271) III; efn-4 (luc242), vab-2 (luc244), hst-1 (luc261), efn-2 (luc246) IV; hst-3.2 (luc215), hst-6 (luc218), hst-2 (luc263) X; lucEx1399 (tph-1::gfp, ttx-3::mcherry*	luc215-275: miR-51Fam seed recognition element replaced for the lsy-6 binding site (*66*), modified to include a BsiWI restriction site (CGtacgTTATACAAAA). lucEx1399: tph-1p::gfp, ttx-3::mcherry. Outcrossed isolate 1	This study.
MLC2892	*mig-1 (luc272), kal-1 (luc216) I; eff-1 (luc259) II; lon-1 (luc268), cdh-3 (luc275) lin-12 (luc271) III; efn-4 (luc242), vab-2 (luc244), hst-1 (luc261), efn-2 (luc246) IV; hst-3.2 (luc215), hst-6 (luc218), hst-2 (luc263) X; lucEx1399 (tph-1::gfp, ttx-3::mcherry*	luc215-275: miR-51Fam seed recognition element replaced for the lsy-6 binding site (*66*), modified to include a BsiWI restriction site (CGtacgTTATACAAAA). lucEx1399: tph-1p::gfp, ttx-3::mcherry. Outcrossed isolate 2	This study.
MLC2893	*mig-1 (luc272), kal-1 (luc216) I; eff-1 (luc259) II; lon-1 (luc268), cdh-3 (luc275) lin-12 (luc271) III; efn-4 (luc242), vab-2 (luc244), hst-1 (luc261), efn-2 (luc246) IV; hst-3.2 (luc215), hst-6 (luc218), hst-2 (luc263) X; lucEx1399 (tph-1::gfp, ttx-3::mcherry*	luc215-275: miR-51Fam seed recognition element replaced for the lsy-6 binding site (*66*), modified to include a BsiWI restriction site (CGtacgTTATACAAAA). lucEx1399: tph-1p::gfp, ttx-3::mcherry. Outcrossed isolate 3	This study.

Other Supplementary Materials

Movie S1. (separate file)
Embryogenesis of a wild-type embryo imaged under Nomarski optics from mid gastrulation until hatching, about 10 hours. Playback speed is ~700x the normal developmental time. Note the first phase of elongation until the "comma" stage, then as twitching begins the muscle-driven phase of elongation stretches the embryo to its final length. *C. elegans* embryos are 50x30 µm.

Movie S2. (separate file)
Embryogenesis of three embryos lacking miR-51^{Fam} imaged under Nomarski optics from mid gastrulation for about 10 hours. Playback speed is ~700x the normal developmental time. Note the first phase of elongation until the "comma" stage proceeds seemingly normally. Twitching begins, suggesting muscles are active, but the second phase of elongation fails to fully stretch the embryo to its final length. These embryos fail to hatch, representative of 50-60% of *mir-51fam (0)* embryos. *C. elegans* embryos are 50x30 µm.

Data S1. (separate file)
Small RNA sequencing data. Spike-in normalized miRNA quantification (in amoles/µg of total RNA) in comma-stage embryos of different genotypes: N2 (wild type), MLC1800 (miR-51 family null), MLC2290 (miR-54 cluster only), MLC2394 (miR-54, -55 only), MLC2393 (miR-54 only), MLC1997 (pre-mir-52 hairpin endogenously replaced by pre-mir-51 hairpin, all other miR-51fam deleted), MLC1918 (pre-mir-52 hairpin endogenously replaced by pre-mir-57mut hairpin, all other miR-51fam deleted), MLC1917 (pre-mir-52 hairpin endogenously replaced by pre-mir-53 hairpin, all other miR-51fam deleted). For each genotype, independent duplicates were sequenced. We report each replicate independently, as well as an average for each genotype.

Data S2. (separate file)
mRNA sequencing data (Quant-seq). Normalized reads and differential expression analysis between wild type embryos and miR-51 family null embryos at three different stages (190-cells, bean and 2-fold). Each stage is in a separate sheet in the Excel workbook. Two independent duplicates for each genotype are reported. Log2FC and p values reported are from DE-seq analysis.

REFERENCES AND NOTES

1. C. Alberti, L. Cochella, A framework for understanding the roles of miRNAs in animal development. *Development* **144**, 2548–2559 (2017).

2. L. F. Sempere, C. N. Cole, M. A. McPeek, K. J. Peterson, The phylogenetic distribution of metazoan microRNAs: Insights into evolutionary complexity and constraint. *J. Exp. Zool. B Mol. Dev. Evol.* **306**, 575–588 (2006).

3. A. M. Heimberg, A. M. Heimberg, L. F. Sempere, L. F. Sempere, V. N. Moy, V. N. Moy, P. C. J. Donoghue, P. C. J. Donoghue, K. J. Peterson, K. J. Peterson, MicroRNAs and the advent of vertebrate morphological complexity. *Proc. Natl. Acad. Sci. U.S.A.* **105**, 2946–2950 (2008).

4. A. Grimson, M. Srivastava, B. Fahey, B. J. Woodcroft, H. R. Chiang, N. King, B. M. Degnan, D. S. Rokhsar, D. P. Bartel, Early origins and evolution of microRNAs and Piwi-interacting RNAs in animals. *Nature* **455**, 1193–1197 (2008).

5. B. Fromm, D. Domanska, E. Høye, V. Ovchinnikov, W. Kang, E. Aparicio-Puerta, M. Johansen, K. Flatmark, A. Mathelier, E. Hovig, M. Hackenberg, M. R. Friedländer, K. J. Peterson, MirGeneDB 2.0: The metazoan microRNA complement. *Nucleic Acids Res.* **48**, D132–D141 (2020).

6. A. W. Clarke, E. Høye, A. A. Hembrom, V. M. Paynter, J. Vinther, Ł. Wyrożemski, I. Biryukova, A. Formaggioni, V. Ovchinnikov, H. Herlyn, A. Pierce, C. Wu, M. Aslanzadeh, J. Cheneby, P. Martinez, M. R. Friedländer, E. Hovig, M. Hackenberg, S. U. Umu, M. Johansen, K. J. Peterson, B. Fromm, MirGeneDB 3.0: Improved taxonomic sampling, uniform nomenclature of novel conserved microRNA families and updated covariance models. *Nucleic Acids Res.* **53**, D116–D128 (2025).

7. N. S. Sokol, P. Xu, Y. N. Jan, V. Ambros, Drosophila let-7 microRNA is required for remodeling of the neuromusculature during metamorphosis. *Genes Dev.* **22**, 1591–1596 (2008).

8. E. Alvarez-Saavedra, H. R. Horvitz, Many families of *C. elegans* microRNAs are not essential for development or viability. *Curr. Biol.* **20**, 367–373 (2010).

9. W. R. Shaw, J. Armisen, N. J. Lehrbach, E. A. Miska, The conserved miR-51 microRNA family is redundantly required for embryonic development and pharynx attachment in *Caenorhabditis elegans*. *Genetics* **185**, 897–905 (2010).

10. J. Brennecke, A. Stark, R. B. Russell, S. M. Cohen, Principles of microRNA–target recognition. *PLoS Biol.* **3**, e85 (2005).

11. A. Grimson, K. K.-H. Farh, W. K. Johnston, P. Garrett-Engele, L. P. Lim, D. P. Bartel, MicroRNA targeting specificity in mammals: Determinants beyond seed pairing. *Mol. Cell* **27**, 91–105 (2007).

12. J. P. Broughton, M. T. Lovci, J. L. Huang, G. W. Yeo, A. E. Pasquinelli, Pairing beyond the seed supports microRNA targeting specificity. *Mol. Cell* **64**, 320–333 (2016).

13. R. J. Parchem, J. Ye, R. L. Judson, M. F. LaRussa, R. Krishnakumar, A. Blelloch, M. C. Oldham, R. Blelloch, Two miRNA clusters reveal alternative paths in late-stage reprogramming. *Cell Stem Cell* **14**, 617–631 (2014).

14. K. Chen, N. Rajewsky, The evolution of gene regulation by transcription factors and microRNAs. *Nat. Rev. Genet.* **8**, 93–103 (2007).

15. S. Lutzmayer, B. Enugutti, M. D. Nodine, Novel small RNA spike-in oligonucleotides enable absolute normalization of small RNA-Seq data. *Sci. Rep.* **7**, 5913 (2017).

16. P. J. Dexheimer, J. Wang, L. Cochella, Two microRNAs are sufficient for embryonic patterning in *C. elegans*. *Curr. Biol.* **30**, 5058–5065.e5 (2020).

17. N. J. Martinez, M. C. Ow, J. S. Reece-Hoyes, M. I. Barrasa, V. R. Ambros, A. J. M. Walhout, Genome-scale spatiotemporal analysis of *Caenorhabditis elegans* microRNA promoter activity. *Genome Res.* **18**, 2005–2015 (2008).

18. N. J. Martinez, R. I. Gregory, Argonaute2 expression is post-transcriptionally coupled to microRNA abundance. *RNA* **19**, 605–612 (2013).

19. B. Reichholf, V. A. Herzog, N. Fasching, R. A. Manzenreither, I. Sowemimo, S. L. Ameres, Time-resolved small RNA sequencing unravels the molecular principles of microRNA homeostasis. *Mol. Cell* **75**, 756–768.e7 (2019).

20. B. P. Lewis, C. B. Burge, D. P. Bartel, Conserved seed pairing, often flanked by adenosines, indicates that thousands of human genes are microRNA targets. *Cell* **120**, 15–20 (2005).

21. B. J. Reinhart, F. J. Slack, M. Basson, A. E. Pasquinelli, J. C. Bettinger, A. E. Rougvie, H. R. Horvitz, G. Ruvkun, The 21-nucleotide let-7 RNA regulates developmental timing in *Caenorhabditis elegans*. *Nature* **403**, 901–906 (2000).

22. B. R. M. Schulman, A. Esquela-Kerscher, F. J. Slack, Reciprocal expression of lin-41 and the microRNAs let-7 and mir-125 during mouse embryogenesis. *Dev. Dyn.* **234**, 1046–1054 (2005).

23. Y.-C. Lin, L.-C. Hsieh, M.-W. Kuo, J. Yu, H.-H. Kuo, W.-L. Lo, R.-J. Lin, A. L. Yu, W.-H. Li, Human TRIM71 and its nematode homologue are targets of let-7 microRNA and its zebrafish orthologue is essential for development. *Mol. Biol. Evol.* **24**, 2525–2534 (2007).

24. P. Gutiérrez-Pérez, E. M. Santillán, T. Lendl, J. Wang, A. Schrempf, T. L. Steinacker, M. Asparuhova, M. Brandstetter, D. Haselbach, L. Cochella, miR-1 sustains muscle physiology by controlling V-ATPase complex assembly. *Sci. Adv.* **7**, eabh1434 (2021).

25. V. Agarwal, G. W. Bell, J.-W. Nam, D. P. Bartel, Predicting effective microRNA target sites in mammalian mRNAs. *eLife* **4**, 101 (2015).

26. J. S. Simske, J. Hardin, Getting into shape: Epidermal morphogenesis in *Caenorhabditis elegans* embryos. *Bioessays* **23**, 12–23 (2001).

27. E. I. Rugarli, E. Di Schiavi, M. A. Hilliard, S. Arbucci, C. Ghezzi, A. Facciolli, G. Coppola, A. Ballabio, P. Bazzicalupo, The Kallmann syndrome gene homolog in *C. elegans* is involved in epidermal morphogenesis and neurite branching. *Development* **129**, 1283–1294 (2002).

28. H. E. Bülow, K. L. Berry, L. H. Topper, E. Peles, O. Hobert, Heparan sulfate proteoglycan-dependent induction of axon branching and axon misrouting by the Kallmann syndrome gene kal-1. *Proc. Natl. Acad. Sci. U.S.A.* **99**, 6346–6351 (2002).

29. C. A. Díaz-Balzac, M. I. Lázaro-Peña, G. A. Ramos-Ortiz, H. E. Bülow, The adhesion molecule KAL-1/anosmin-1 regulates neurite branching through a SAX-7/L1CAM-EGL-15/FGFR receptor complex. *Cell Rep.* **11**, 1377–1384 (2015).

30. M. L. Hudson, T. Kinnunen, H. N. Cinar, A. D. Chisholm, *C. elegans* Kallmann syndrome protein KAL-1 interacts with syndecan and glypican to regulate neuronal cell migrations. *Dev. Biol.* **294**, 352–365 (2006).

31. A. Sebé-Pedrós, B. M. Degnan, I. Ruiz-Trillo, The origin of Metazoa: A unicellular perspective. *Nat. Rev. Genet.* **18**, 498–512 (2017).

32. X. Lin, Functions of heparan sulfate proteoglycans in cell signaling during development. *Development* **131**, 6009–6021 (2004).

33. E. Tecle, C. A. Diaz-Balzac, H. E. Bülow, Distinct 3-O-sulfated heparan sulfate modification patterns are required for kal-1-dependent neurite branching in a context-dependent manner in *Caenorhabditis elegans*. *G3* **3**, 541–552 (2013).

34. L. S. Babonis, M. Q. Martindale, Phylogenetic evidence for the modular evolution of metazoan signalling pathways. *Philos. Trans. R. Soc. B Biol. Sci.* **372**, 20150477 (2017).

35. M. Labouesse, Role of the extracellular matrix in epithelial morphogenesis: A view from *C. elegans*. *Organogenesis* **8**, 65–70 (2012).

36. D. C. Merz, G. Alves, T. Kawano, H. Zheng, J. G. Culotti, UNC-52/perlecan affects gonadal leader cell migrations in *C. elegans* hermaphrodites through alterations in growth factor signaling. *Dev. Biol.* **256**, 173–186 (2003).

37. T. Kinnunen, Z. Huang, J. Townsend, M. M. Gatdula, J. R. Brown, J. D. Esko, J. E. Turnbull, Heparan 2-O-sulfotransferase, hst-2, is essential for normal cell migration in *Caenorhabditis elegans*. *Proc. Natl. Acad. Sci. U.S.A.* **102**, 1507–1512 (2005).

38. T. T. K. Vuong-Brender, X. Yang, M. Labouesse, *C. elegans* embryonic morphogenesis. *Curr. Top. Dev. Biol.* **116**, 597–616 (2016).

39. J. M. Bosher, B.-S. Hahn, R. Legouis, S. Sookhareea, R. M. Weimer, A. Gansmuller, A. D. Chisholm, A. M. Rose, J.-L. Bessereau, M. Labouesse, The *Caenorhabditis elegans* vab-10 spectraplakin isoforms protect the epidermis against internal and external forces. *J. Cell Biol.* **161**, 757–768 (2003).

40. H. Zahreddine, H. Zhang, M. Diogon, Y. Nagamatsu, M. Labouesse, CRT-1/calreticulin and the E3 ligase EEL-1/HUWE1 control hemidesmosome maturation in *C. elegans* development. *Curr. Biol.* **20**, 322–327 (2010).

41. A. Vasquez-Rifo, G. Jannot, J. Armisen, M. Labouesse, S. I. A. Bukhari, E. L. Rondeau, E. A. Miska, M. J. Simard, Developmental characterization of the microRNA-specific *C. elegans* Argonautes *alg*-1 and *alg*-2. *PLOS ONE* **7**, e33750–11 (2012).

42. C. Desai, G. Garriga, S. L. McIntire, H. R. Horvitz, A genetic pathway for the development of the *Caenorhabditis elegans* HSN motor neurons. *Nature* **336**, 638–646 (1988).

43. C. Rhiner, S. Gysi, E. Fröhli, M. O. Hengartner, A. Hajnal, Syndecan regulates cell migration and axon guidance in *C. elegans*. *Development* **132**, 4621–4633 (2005).

44. M. E. Pedersen, G. Snieckute, K. Kagias, C. Nehammer, H. A. B. Multhaupt, J. R. Couchman, R. Pocock, An epidermal microRNA regulates neuronal migration through control of the cellular glycosylation state. *Science* **341**, 1404–1408 (2013).

45. T. K. Kinnunen, Combinatorial roles of heparan sulfate proteoglycans and heparan sulfates in *Caenorhabditis elegans* neural development. *PLOS ONE* **9**, e102919 (2014).

46. K. Saied-Santiago, R. A. Townley, J. D. Attonito, D. S. da Cunha, C. A. Díaz-Balzac, E. Tecle, H. E. Bülow, Coordination of heparan sulfate proteoglycans with Wnt signaling to control cellular migrations and positioning in *Caenorhabditis elegans*. *Genetics* **206**, 1951–1967 (2017).

47. Y. Iosilevskii, B. Podbilewicz, Programmed cell fusion in development and homeostasis. *Curr. Top. Dev. Biol.* **144**, 215–244 (2021).

48. Y. Yang, Y. Zhang, W.-J. Li, Y. Jiang, Z. Zhu, H. Hu, W. Li, J.-W. Wu, Z.-X. Wang, M.-Q. Dong, S. Huang, G. Ou, Spectraplakin induces positive feedback between fusogens and the actin cytoskeleton to promote cell-cell fusion. *Dev. Cell* **41**, 107–120.e4 (2017).

49. L. Cochella, O. Hobert, Embryonic priming of a miRNA locus predetermines postmitotic neuronal left/right asymmetry in *C. elegans*. *Cell* **151**, 1229–1242 (2012).

50. B. T. Kasper, S. Koppolu, L. K. Mahal, Insights into miRNA regulation of the human glycome. *Biochem. Biophys. Res. Commun.* **445**, 774–779 (2014).

51. D. M. Kasper, J. Hintzen, Y. Wu, J. J. Ghersi, H. K. Mandl, K. E. Salinas, W. Armero, Z. He, Y. Sheng, Y. Xie, D. W. Heindel, E. J. Park, W. C. Sessa, L. K. Mahal, C. Lebrilla, K. K. Hirschi, S. Nicoli, The N-glycome regulates the endothelial-to-hematopoietic transition. *Science* **370**, 1186–1191 (2020).

52. A. Sowińska-Seidler, M. Piwecka, E. Olech, M. Socha, A. Latos-Bieleńska, A. Jamsheer, Hyperosmia, ectrodactyly, mild intellectual disability, and other defects in a male patient with an X-linked partial microduplication and overexpression of the KAL1 gene. *J. Appl. Genet.* **56**, 177–184 (2015).

53. A. J. Giraldez, R. M. Cinalli, M. E. Glasner, A. J. Enright, J. M. Thomson, S. Baskerville, S. M. Hammond, D. P. Bartel, A. F. Schier, MicroRNAs regulate brain morphogenesis in zebrafish. *Science* **308**, 833–838 (2005).

54. R. J. Parchem, N. Moore, J. L. Fish, J. G. Parchem, T. T. Braga, A. Shenoy, M. C. Oldham, J. L. R. Rubenstein, R. A. Schneider, R. Blelloch, miR-302 is required for timing of neural differentiation, neural tube closure, and embryonic viability. *Cell Rep.* **12**, 760–773 (2015).

55. A. L. Abbott, E. Alvarez-Saavedra, E. A. Miska, N. C. Lau, D. P. Bartel, H. R. Horvitz, V. Ambros, The let-7 microRNA family members mir-48, mir-84, and mir-241 function together to regulate developmental timing in *Caenorhabditis elegans*. *Dev. Cell* **9**, 403–414 (2005).

56. S. Kataruka, M. Modrak, V. Kinterova, R. Malik, D. M. Zeitler, F. Horvat, J. Kanka, G. Meister, P. Svoboda, MicroRNA dilution during oocyte growth disables the microRNA pathway in mammalian oocytes. *Nucleic Acids Res.* **48**, 8050–8062 (2020).

57. M. N. McCall, M.-S. Kim, M. Adil, A. H. Patil, Y. Lu, C. J. Mitchell, P. Leal-Rojas, J. Xu, M. Kumar, V. L. Dawson, T. M. Dawson, A. S. Baras, A. Z. Rosenberg, D. E. Arking, K. H. Burns, A. Pandey, M. K. Halushka, Toward the human cellular microRNAome. *Genome Res.* **27**, 1769–1781 (2017).

58. S. Brenner, The genetics of *Caenorhabditis elegans*. *Genetics* **77**, 71–94 (1974).

59. A. Paix, A. Folkmann, D. Rasoloson, G. Seydoux, High efficiency, homology-directed genome editing in *Caenorhabditis elegans* using CRISPR-Cas9 ribonucleoprotein complexes. *Genetics* **201**, 47–54 (2015).

60. S. R. Sternberg, Biomedical image processing. *Computertomographie* **16**, 22–34 (1983).

61. T. Smith, A. Heger, I. Sudbery, UMI-tools: Modeling sequencing errors in unique molecular identifiers to improve quantification accuracy. *Genome Res.* **27**, 491–499 (2017).

62. A. Dobin, C. A. Davis, F. Schlesinger, J. Drenkow, C. Zaleski, S. Jha, P. Batut, M. Chaisson, T. R. Gingeras, STAR: Ultrafast universal RNA-seq aligner. *Bioinformatics* **29**, 15–21 (2013).

63. S. Anders, P. T. Pyl, W. Huber, HTSeq—A Python framework to work with high-throughput sequencing data. *Bioinformatics* **31**, 166–169 (2015).

64. M. I. Love, W. Huber, S. Anders, Moderated estimation of fold change and dispersion for RNA-seq data with DESeq2. *Genome Biol.* **15**, 550 (2014).

65. S. G. Clark, C. Chiu, *C. elegans* ZAG-1, a Zn-finger-homeodomain protein, regulates axonal development and neuronal differentiation. *Development* **130**, 3781–3794 (2003).

66. D. Didiano, O. Hobert, Perfect seed pairing is not a generally reliable predictor for miRNA-target interactions. *Nat. Struct. Mol. Biol.* **13**, 849–851 (2006).
